# Seven new species of *Cichlidogyrus* Paperna, 1960 (Monogenea: Dactylogyridae) parasitizing the gills of Congolese cichlids from northern Lake Tanganyika

**DOI:** 10.7717/peerj.5604

**Published:** 2018-10-23

**Authors:** Chahrazed Rahmouni, Maarten P.M. Vanhove, Andrea Šimková

**Affiliations:** 1 Department of Botany and Zoology, Faculty of Science, Masaryk University, Brno, Czech Republic; 2 Zoology Unit, Finnish Museum of Natural History, University of Helsinki, Helsinki, Finland; 3 Laboratory of Biodiversity and Evolutionary Genomics, Department of Biology, University of Leuven, Leuven, Belgium; 4 Centre for Environmental Sciences, Research Group Zoology: Biodiversity and Toxicology, Universiteit Hasselt, Diepenbeek, Belgium

**Keywords:** Congo, Cichlidae, *Interochromis*, *Petrochromis*, *Callochromis*, Platyhelminthes, *Tylochromis*, *C. koblmuelleri* sp. nov., *C. habluetzeli* sp. nov., *C. masilyai* sp. nov., *C. antoineparisellei* sp. nov., *Cardiopharynx*, *C. sergemorandi* sp. nov., *Cyphotilapia*, *C. adkoningsi* sp. nov., *Xenotilapia*, *C. salzburgeri* sp. nov.

## Abstract

Seven new species of *Cichlidogyrus* Paperna, 1960 (Monogenea: Dactylogyridae) isolated from the gills of six cichlid host species belonging to four tribes and sampled from the Congolese coastline of Lake Tanganyika (LT) are described: *Cichlidogyrus adkoningsi* sp. nov. from *Cyphotilapia frontosa* (tribe Cyphotilapiini); *C. koblmuelleri* sp. nov. from *Cardiopharynx schoutedeni* (Ectodini); *C. habluetzeli* sp. nov. from *C. schoutedeni* and *C. frontosa*; *C. antoineparisellei* sp. nov. from *Interochromis loocki* (Tropheini); *C. masilyai* sp. nov. from *Petrochromis orthognathus* (Tropheini); *C. salzburgeri* sp. nov. from *P. trewavasae*, and *C. sergemorandi* sp. nov. from *Tylochromis polylepis* (Tylochromini). This study represents the first parasitological examination of cyphotilapiine cichlid hosts. Representatives of the Tanganyikan ectodine, tropheine, and tylochromine cichlids previously sampled from various localities in the lake yielded nine, twelve, and two described species of *Cichlidogyrus*, respectively. The study further includes a morphological characterization of the male copulatory organ of six undescribed species of *Cichlidogyrus* found on the gills of the tropheines *I. loocki* and *P. orthognathus,* and on those of *Callochromis melanostigma* and *Xenotilapia flavipinnis* (both Ectodini). Geographical variation in the monogenean fauna of *I. loocki* was observed. The most closely related cichlid species investigated in this study harboured *Cichlidogyrus* spp. exhibiting some similarities in their sclerotized structures. Thus, our paper provides additional evidence of the high species richness of *Cichlidogyrus* and the link with their hosts’s phylogenetic affinities in LT.

## Introduction

With an estimated 3,000 species distributed from Central and South America, across Africa to Madagascar, and to the Middle East and the Indian subcontinent (Chakrabarty, 2004), cichlid fishes represent one of the most species-rich families of vertebrates, accounting for about 10% of total teleost diversity (Takahashi & Koblmüller, 2011; Wanek & Sturmbauer, 2015). The Great African Rift Lakes Malawi, Victoria, and Tanganyika harbour cichlid flocks exhibiting high morphological, ecological, and genetic diversity (Takahashi & Sota, 2016). The exact number of species inhabiting these three lakes is still unknown, but approximately 2,000 species have been described (Koblmüller, Sefc & Sturmbauer, 2008). Lake Tanganyika (LT), located in the Great Rift Valley in central East Africa, is the deepest and oldest lake in Africa (Cohen et al., 1997) and the second deepest and oldest lake in the world (Salzburger et al., 2005). It holds the most diverse cichlid assemblages, comprised of several lineages of mostly endemic species classified into more than 50 genera and 12–14 tribes (Snoeks, 2000; Koblmüller, Sefc & Sturmbauer, 2008; Takahashi & Sota, 2016). Over 250 cichlid species are known to inhabit this lake (Takahashi & Koblmüller, 2011). Cichlids represent a textbook model in evolutionary biology (Kocher, 2004). Their mechanisms of speciation by rapid radiation make them crucial to the study of biological diversification, dynamics, and functions (Barluenga & Meyer, 2010; Takahashi & Koblmüller, 2011). Cichlid monogeneans are a promising tool for elucidating the speciation of both fish and parasites (Vanhove et al., 2015, 2016).

Among the 14 monogenean parasite genera known to infect cichlids, six (*Urogyrus* Bilong Bilong, Birgi & Euzet, 1994; *Enterogyrus* Paperna, 1963; *Onchobdella* Paperna, 1968; *Scutogyrus* Pariselle & Euzet, 1995; *Cichlidogyrus* (Dactylogyridae Bychowski, 1933), and *Gyrodactylus* von Nordmann, 1832 (Gyrodactylidae Van Beneden & Hesse, 1863)) were reported from African cichlids (Pariselle & Euzet, 2009; Pariselle et al., 2011; Mendoza-Palmero et al., 2017). More than 100 African and Levantine cichlid species have been investigated for the presence of monogenean parasites (Pariselle & Euzet, 2009; Vanhove et al., 2016). *Cichlidogyrus* Paperna, 1960 is the most species-rich genus and is mostly restricted to African and Levantine hosts (a few species were isolated from non-cichlid hosts, see for instance Birgi & Lambert (1986)) (Pariselle & Euzet, 2009). To date, 111 valid species of *Cichlidogyrus* have been recognized in African cichlids (see the overview of Tanganyikan and non-Tanganyikan species of *Cichlidogyrus* published recently by Rahmouni et al. (2017)). Some Tanganyikan cichlid tribes remain to be investigated for their gill flatworms.

No parasitological data are available on the Cyphotilapiini Salzburger et al., 2002 with its three endemic representatives *Cyphotilapia frontosa* (Boulenger, 1906), *Cyphotilapia gibberosa* (Takahashi & Nakaya, 2003), and *Trematochromis benthicola* (Matthes, 1962) (Muschick, Indermaur & Salzburger, 2012; Meyer, Matschiner & Salzburger, 2015; Takahashi & Sota, 2016). Among the 34 valid cichlid species that belong to the endemic Tanganyikan tribe Ectodini Poll, 1986, which includes 10 genera, only four species were studied for the presence of parasites and nine *Cichlidogyrus* spp. were described. Vanhove, Volckaert & Pariselle (2011) described four *Cichlidogyrus* spp. (*C. centesimus, C. makasai*, and *C. vandekerkhovei* Vanhove, Volckaert & Pariselle, 2011 on Congolese and Zambian *Ophthalmotilapia ventralis* (Boulenger, 1898) and Tanzanian *O. boops* (Boulenger, 1901) and *O. nasuta* (Poll & Matthes, 1962), and *C. sturmbaueri* Vanhove, Volckaert & Pariselle, 2011 on Zambian *O. ventralis* and Tanzanian *O. nasuta*). Later, Rahmouni et al. (2017) investigated *Aulonocranus dewindti* (Boulenger, 1899) and *O. nasuta* from the Burundese part of LT and described two *Cichlidogyrus* spp. on *A. dewindti* (*C. discophonum* and *C. pseudoaspiralis* Rahmouni, Vanhove & Šimková, 2017), and three species on *O. nasuta* (*C. aspiralis*, *C. glacicremoratus* and *C. rectangulus* Rahmouni, Vanhove & Šimková, 2017).

*Cardiopharynx* Poll, 1942 is monotypic and represented by *C. schoutedeni* Poll, 1942 (Konings, 2015). Still in the Ectodini, *Callochromis* Regan, 1920 consists of three nominal species: *Callochromis macrops* (Boulenger, 1898), *C. melanostigma* (Boulenger, 1906), and *C. pleurospilus* (Boulenger, 1906) (Konings, 2015), whereas *Xenotilapia* Boulenger, 1898 includes 13–17 species (Kidd et al., 2012). *Xenotilapia flavipinnis* Poll, 1985 has a lake-wide distribution (Konings, 2015). In contrast to some other members of the Ectodini, there are no data available on the parasite fauna hosted by representatives of *Callochromis* and *Xenotilapia.*

The Tropheini is one of the most species-rich cichlid tribes endemic to LT with nine genera including approximately 24 species (Takahashi & Koblmüller, 2014). At least eight species of *Petrochromis* Boulenger, 1898, a representative of the Tropheini, have been described (Sturmbauer et al., 2003; Takahashi & Sota, 2016). *Petrochromis orthognathus* Matthes, 1959 is restricted to the northern two-thirds of the lake. *Petrochromis trewavasae* Poll, 1948 is found in the southern part of the lake, usually in sympatry with *Petrochromis ephippium* Brichard, 1989, a morphologically similar species considered conspecific to *P. trewavasae* (Konings, 2015). *Interochromis* Yamaoka, Hori & Kuwamura, 1998 is a monotypic genus erected because of the morphological and ecological similarities between *I. loocki* and species of *Petrochromis*, and the differences between *I. loocki* and species of the tropheine *Simochromis* Boulenger, 1898 (see overview in Pariselle et al., 2015b). Several studies have been carried out on the parasitic flatworms of these cichlids. Gillardin et al. (2012) described three *Cichlidogyrus* spp. (*C. steenbergei* and *C. irenae* Gillardin et al., 2012 from Zambian and Congolese *Limnotilapia dardennii* (Boulenger, 1899) and *‘Gnathochromis’ pfefferi* (Boulenger, 1898), respectively, and *C. gistelincki* Gillardin et al., 2012 from Congolese, Tanzanian, and Zambian ‘*Ctenochromis’ horei* (Günther, 1894)). Then, Pariselle et al. (2015b) examined Zambian *I. loocki* and described three *Cichlidogyrus* spp. (*C. buescheri*, *C. schreyenbrichardorum*, and *C. vealli* Pariselle & Vanhove, 2015). In the same study, they compared the haptoral structures of representatives of *Cichlidogyrus* infecting *I. loocki* with those observed in some undescribed *Cichlidogyrus* spp. isolated from representatives of *Petrochromis*. The same team described six species of *Cichlidogyrus* infecting Congolese, Tanzanian and Zambian tropheine cichlids (*C. banyankimbonai*, *C. muterezii, and C. raeymaekersi* Pariselle & Vanhove, 2015 on *Simochromis diagramma* (Günther, 1894); *C. georgesmertensi* Pariselle & Vanhove, 2015 on *Pseudosimochromis babaulti* (Pellegrin, 1927); *C. franswittei* Pariselle & Vanhove, 2015 on *P. marginatus* (Poll, 1956) and *P. curvifrons* (Poll, 1942); and finally *C. frankwillemsi* Pariselle & Vanhove, 2015 on *P. curvifrons* (Van Steenberge et al., 2015)).

Lake Tanganyika harbours representatives of a few non-endemic tribes resulting from colonisation from the lacustrine environment. This is the case of Tylochromini Poll, 1986 with *Tylochromis polylepis* (Boulenger, 1900) as its sole representative in the lake. Members of *Tylochromis* Regan, 1920 inhabit rivers, lakes, and coastal lagoons throughout central and western Africa. Muterezi Bukinga et al. (2012) studied the gill monogeneans of *T. polylepis* and described two species of *Cichlidogyrus* from Congolese host specimens, that is, *C. mulimbwai* and *C. muzumanii* Muterezi Bukinga et al., 2012. In addition, they presented drawings of the hard parts (haptor and reproductive organs) of an undescribed species referred to as *Cichlidogyrus* sp. ‘*T. polylepis* 3’.

The aim of this paper is to study gill monogenean diversity in cichlids belonging to four tribes from the Congolese lakeshore of northern LT. We describe seven new species of *Cichlidogyrus* and provide the morphological characterization of six undescribed congeners.

## Material and Methods

**Figure 1:**
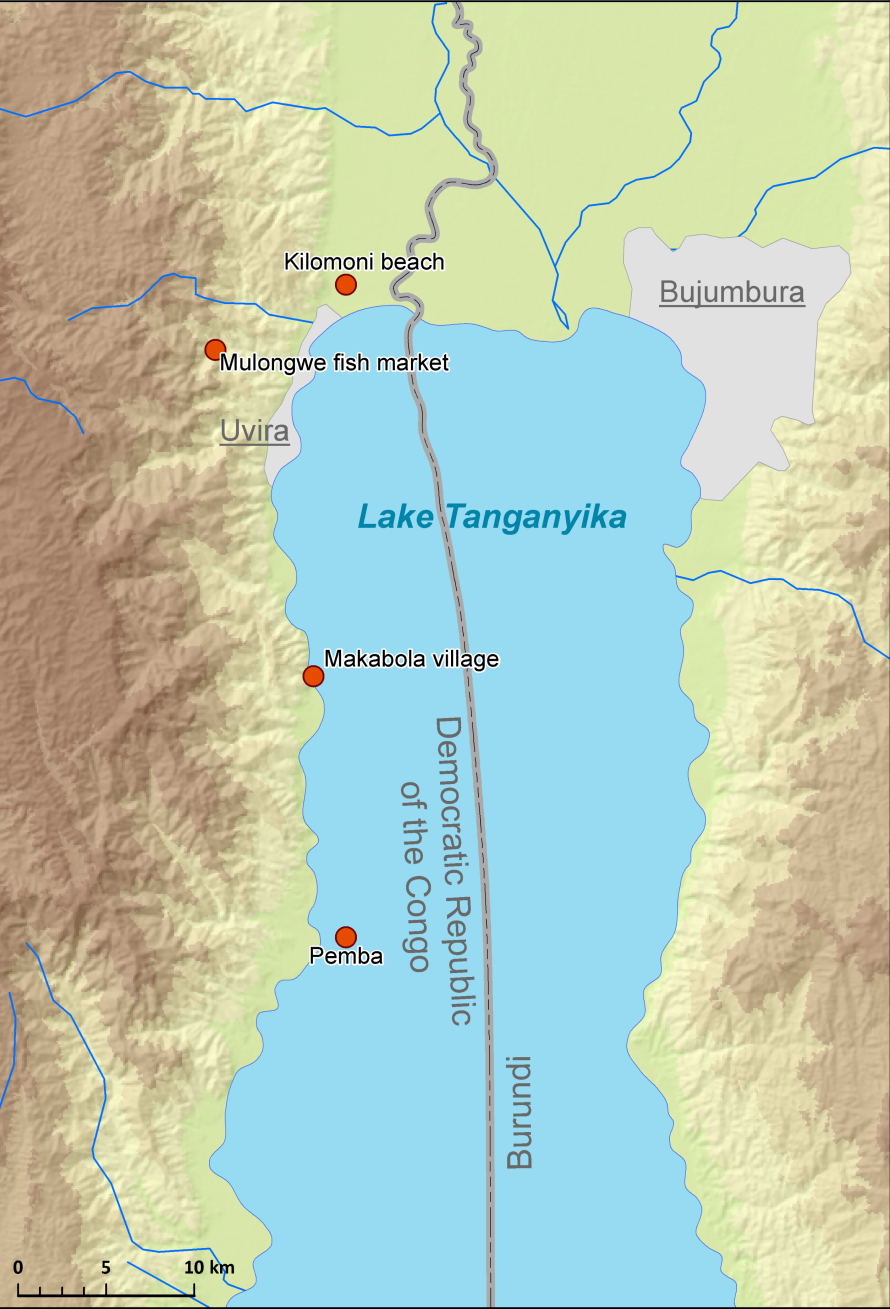
Map of sampling localities in LT.

Cichlid specimens were obtained in September 2013 and August 2016 from LT along the shoreline of the Democratic Republic of the Congo (DRC) (Fig. 1). In total, 26 fish specimens belonging to eight cichlid species of four tribes were purchased from fish markets or captured using gill nets during snorkelling or diving. Fish were placed in a cool box containing ice, transported to the laboratory, and dissected immediately.

Cichlid hosts were identified in situ on the basis of morphological characters by Walter Salzburger (Zoological Institute, University of Basel, Switzerland), Donatien Muzumani Risasi (Centre de Recherche en Hydrobiologie, Uvira, DRC), and Maarten Van Steenberge (the Royal Museum for Central Africa (MRAC), Tervuren and the Royal Belgian Institute of Natural Sciences, Brussels, Belgium). We performed molecular analysis on the samples, and obtained sequence data from the partial cytochrome *b* (cyt-*b*) mitochondrial gene to confirm the identity of the investigated cichlids. Fin clips from the cichlid specimens were preserved in 96% ethanol. Cichlid DNA was extracted using the DNeasy Tissue kit (Qiagen, Hilden, Germany) according to the manufacturer’s protocol. The partial cyt-*b* gene was amplified following Mendlová et al. (2012). The PCR products were loaded onto a 1% agarose gel and subsequently purified, and sequencing was performed following Rahmouni et al. (2017). Nucleotide sequences were edited using Sequencher software v. 5.0 (Gene Codes, Ann Arbor, MI, USA). The identification of cichlid species based on the sequence similarity approach was carried out using the Basic Local Alignment Search Tool (https://blast.ncbi.nlm.nih.gov/Blast.cgi: blastn, default settings), available through the website of the National Centre for Biotechnology Information (NCBI Resource Coordinators, 2017). The newly generated sequences were deposited in GenBank under the accession numbers MH297985–MH298008.

**Figure 2:**
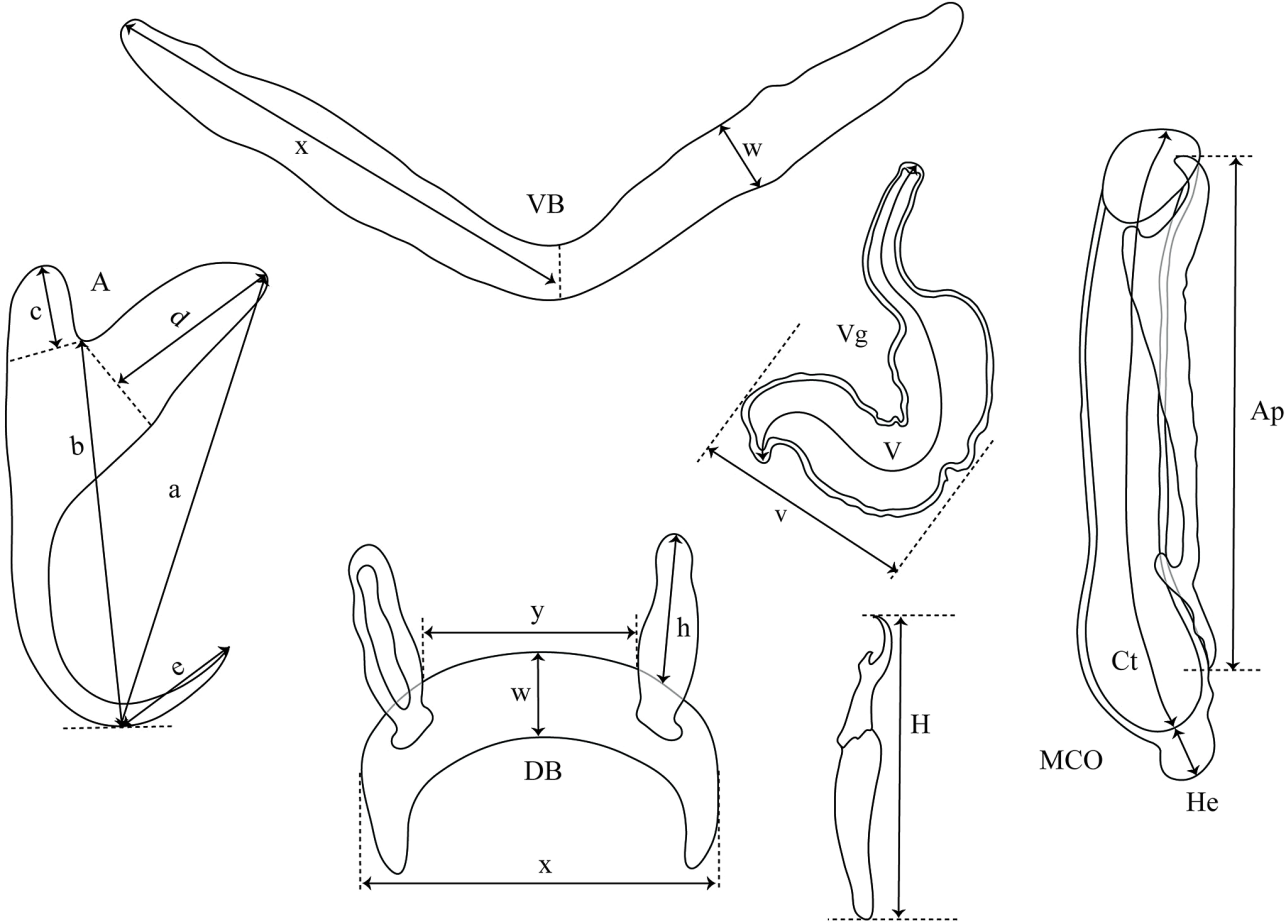
Measurements used in the descriptions of the new species of *Cichlidogyrus.* (A) anchor. (DA) dorsal anchor. (VA) ventral anchor. (a) total length. (b) blade length. (c) shaft length. (d) guard length. (e) point length. (DB) dorsal bar: (h) auricle length. (w) maximum straight width. (x) total length. (y) distance between auricles. (VB) ventral bar: (x) length of one ventral bar branch. (w) maximum width. (H) hook length. (MCO) male copulatory organ straight length. (Ct) copulatory tube curved length. (He) heel straight length. (Ap) accessory piece straight length. (Vg) vagina: (V) vagina total length. (v) vagina width.

Gill arches were separated via dorsal and ventral section using standard parasitological procedures and transferred into a Petri dish containing water. Monogeneans were detached from the gills and isolated according to Musilová, Řehulková & Gelnar (2009) using an MST130 stereoscopic microscope and mounted on slides with glycerine ammonium picrate mixture (GAP) (Malmberg, 1957). Parasite identification was conducted using original descriptions, the systematic revision of dactylogyridean parasites of African cichlids by Pariselle & Euzet (2009), and the recent overview focusing on the genitals of African *Cichlidogyrus* spp. by Rahmouni et al. (2017). Measurements and photographs were taken using an Olympus BX51 phase-contrast microscope and Olympus Stream Image Analysis v. 1.9.3 software. All measurements are included in the species descriptions. They are in micrometres, and are given as the mean followed by the range and the number of measurements (*n*) in parentheses (measurements of some undescribed species are given as the length of the structure in question followed by the number of measurements in parentheses). Drawings of the haptoral sclerotized parts and copulatory organs were made on flattened specimens using an Olympus BX51 microscope equipped with a drawing tube and edited with a graphic tablet compatible with Adobe Illustrator CS6 v. 16.0.0 and Adobe Photoshop v. 13.0. The terminology of haptoral sclerotized parts (anchors and hooks; also termed gripi and uncinuli, respectively) follows Gussev (1983). The numbering of hook pairs (Roman letters I–VII) is that recommended by Mizelle (1936). This method is preferred in adult specimens because it takes into consideration both antero-posterior and dorso–ventral positions of hooks (Kritsky, Thatcher & Boeger, 1986; Řehulková, Mendlová & Šimková, 2013). The lengths of hook pairs (short or long) was assigned following Pariselle & Euzet (2009). The classification of haptoral groups follows Vignon, Pariselle & Vanhove (2011). The metrics used for the hard structures are shown in Fig. 2. The type material was deposited in the Invertebrate collection of the MRAC, Tervuren, Belgium; the Finnish Museum of Natural History (MZH), Helsinki, Finland; and the Muséum National d’Histoire Naturelle (MNHN), Paris, France. Host nomenclature follows FishBase (Froese & Pauly, 2017). The list of museum specimens used for comparison with the new species is presented in Table 1. Sampling was carried out under mission statements 022/MINEURS/CRH-U/2013 and 031/MINRST/CRH-U/2016 from the Centre de Recherche en Hydrobiologie-Uvira. In the absence of relevant animal welfare regulations in the D.R. Congo, the same strict codes of practice enforced within the European Union were applied. This study was approved by the Animal Care and Use Committee of the Faculty of Science, Masaryk University, Brno (Czech Republic), approval nuber CZ01308.

**Table 1:** Comparative museum material examined in the present study.

*Cichlidogyrus* spp.	Host species	Locality	Accession number
*Cichlidogyrus mulimbwai* Muterezi Bukinga et al., 2012	*T. polylepis* (Boulenger, 1900)	Mulembwe & Moba (DRC)	MRAC 37701
*Cichlidogyrus muzumanii* Muterezi Bukinga et al., 2012	*T. polylepis* (Boulenger, 1900)	Mulembwe & Moba (DRC)	MRAC 37699
*Cichlidogyrus buescheri* Pariselle & Vanhove, 2015	*I. loocki* (Poll, 1949)	Kalambo Lodge (Zambia)	MRAC 37744
*Cichlidogyrus schreyenbrichardorum* Pariselle & Vanhove, 2015	*I. loocki* (Poll, 1949)	Kalambo Lodge (Zambia)	MRAC, MRAC 37741
*Cichlidogyrus vealli* Pariselle & Vanhove, 2015	*I. loocki* (Poll, 1949)	Kalambo Lodge (Zambia)	MRAC, MRAC 37743
*Cichlidogyrus discophonum* Rahmouni, Vanhove & Šimková, 2017	*A. dewindti* (Boulenger, 1899)	Nyaruhongoka (Burundi)	MRAC, MRAC 37956
*Cichlidogyrus pseudoaspiralis* Rahmouni, Vanhove & Šimková, 2017	*A. dewindti* (Boulenger, 1899)	Nyaruhongoka (Burundi)	MRAC 37955
*Cichlidogyrus aspiralis* Rahmouni, Vanhove & Šimková, 2017	*O. nasuta* (Poll & Matthes, 1962)	Magara (Burundi)	MRAC 37954

The electronic version of this article in portable document format will constitute a published work according to the International Commission on Zoological Nomenclature (ICZN), and hence the new names contained in the electronic version are effectively published under that Code from the electronic edition alone. This published work and the nomenclatural acts it contains have been registered in ZooBank, the online registration system for the ICZN. The ZooBank LSIDs (Life Science Identifiers) can be resolved, and the associated information viewed through any standard web browser by appending the LSID to the prefix http://zoobank.org/. The LSID for this publication is: urn:lsid:zoobank.org:pub:7076794A-B9EB-4FFC-AC49-66C304EC5BFB. The online version of this work is archived and available from the following digital repositories: PeerJ, PubMed Central, and CLOCKSS.

## Results

### Molecular identification of cichlid hosts

The mitochondrial cytochrome *b* (cyt-*b*) gene fragment of 25 cichlid specimens was successfully amplified. The length of each consensus sequence was 419 bp. The Blast search processed on the NCBI website confirmed the species identification of the cichlid species investigated for the presence of gill parasitic flatworms.

## Species Descriptions

**Figure 3:**
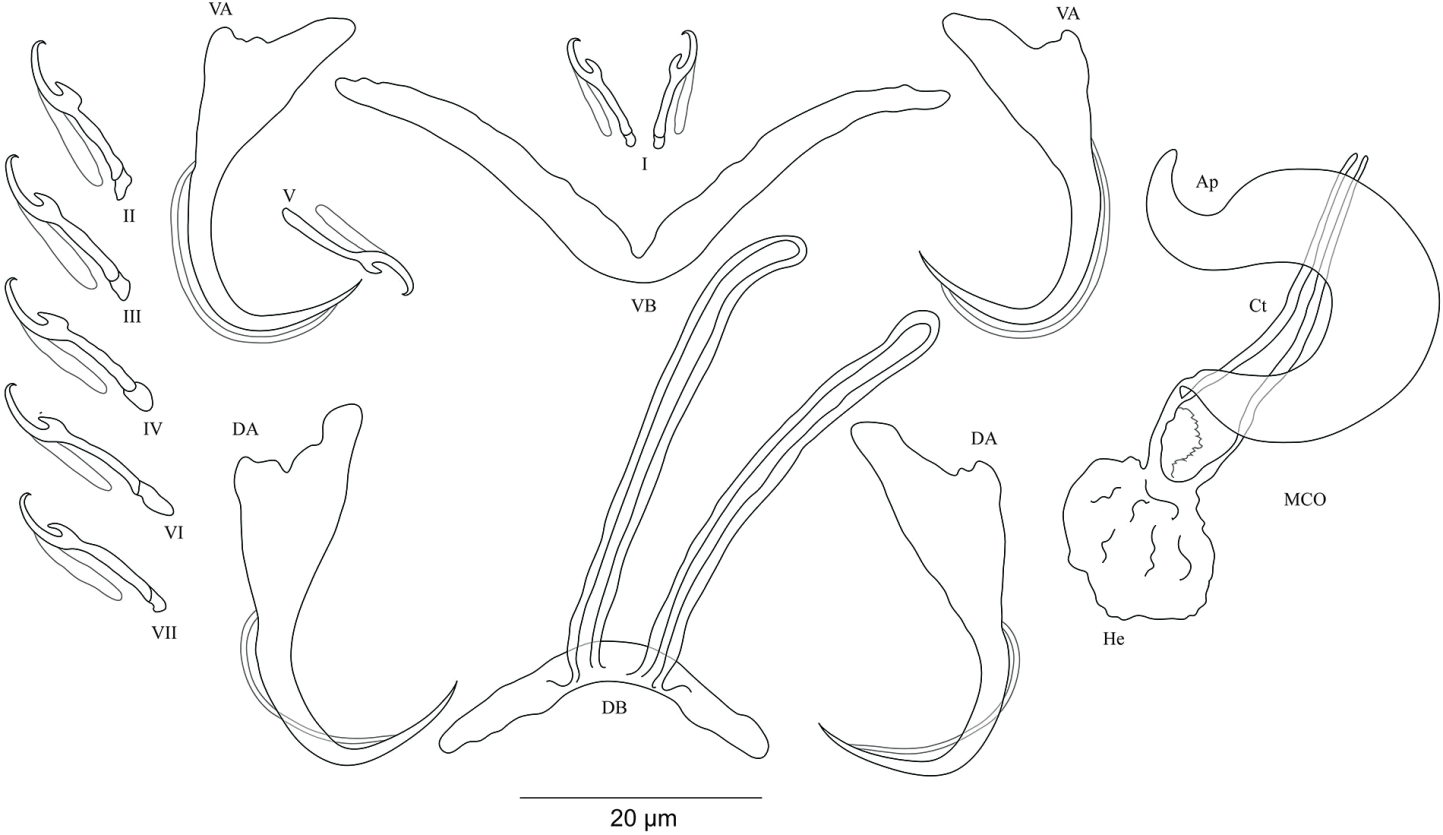
Sclerotized structures of *Cichlidogyrus adkoningsi* sp. nov. ex *Cyphotilapia frontosa.* DA, dorsal anchor; DB, dorsal bar; VA, ventral anchor; VB, ventral bar; I–VII, hooks; MCO, male copulatory organ; Ct, copulatory tube; Ap, accessory piece.

**Dactylogyridae Bychowski, 1933**
***Cichlidogyrus* Paperna, 1960**
***Cichlidogyrus adkoningsi* sp. nov. Fig. 3**
urn:lsid:zoobank.org:act:526C2A74-E3D6-4357-B1E8-03B08B95CE38.

**Description.** Based on three specimens fixed in GAP. Body 516 (482–550; *n* = 3) long, 93 (80–107; *n* = 3) wide at mid-body. Dorsal anchors with short shaft and more pronounced guard and curved blade with arched point: *a* = 31 (30–32; *n* = 3); *b* = 25 (24–26; *n* = 3); *c* = 1–2 (*n* = 3); *d* = 9 (7–10; *n* = 3); *e* = 10 (9–12; *n* = 3). Dorsal bar curved with constant width and relatively long auricles: *h* = 38 (35–44; *n* = 3); *w* = 3–4 (*n* = 3); *x* = 28 (26–30; *n* = 3); *y* = 2 (1–4; *n* = 3). Ventral anchors with shorter shaft than guard, curved blade with arched point: *a* = 28 (26–29; *n* = 3); *b* = 24 (23–25; *n* = 3); *c* = 1–2 (*n* = 3), *d* = 7 (6–8; *n* = 3); *e* = 11 (10–13; *n* = 3). V-shaped ventral bar: *w* = 4 (3–5; *n* = 3); *x* = 32 (29–36; *n* = 3). Haptor with seven short hook pairs, hooks V retain their larval size (*sensu* Pariselle & Euzet, 2003, 2009), each hook with erect thumb and shank comprised of two subunits: pair I = 11 (10–12; *n* = 3) long, pair II = 14–15 (*n* = 3) long, pair III = 15–16 (*n* = 3) long, pair IV = 16–17 (*n* = 3) long, pair V = 11–12 (*n* = 3) long, pair VI = 17–18 (*n* = 3) long, and pair VII = 17 (16–18, *n* = 3) long. Male copulatory organ with relatively short copulatory duct, slightly curved halfway and tapered distally: MCO = 45 (42–47; *n* = 3); Ct = 31 (29–33; *n* = 3). Heel irregularly shaped, He = 10 (9–11; *n* = 3). Accessory piece linked to the basal bulb, C-shaped, thick in the middle part, ending in hook, Ap = 26 (24–28; *n* = 13). Vagina non-sclerotized.

**Diagnosis.** *Cichlidogyrus adkoningsi* sp. nov. belongs to the group of species which exhibit short hook pairs I–IV, VI, and VII (*sensu* Vignon, Pariselle & Vanhove, 2011), a copulatory duct without a swollen proximal portion, and a non-sclerotized vagina (see Pariselle & Euzet, 2003), just like *C. attenboroughi* Kmentová, Gelnar, Koblmüller et al., 2016; *C. banyankimbonai*; *C. berminensis* Pariselle, Bitja Nyom & Bilong Bilong, 2013; *C. bifurcatus* Paperna, 1960; *C. brunnensis* Kmentová, Gelnar, Koblmüller et al., 2016; *C. buescheri*; *C. consobrini* Jorissen, Pariselle & Vanhove, 2018; *C. discophonum*; *C. evikae* Rahmouni, Vanhove & Šimková, 2017; *C. fontanai* Pariselle & Euzet, 1997; *C. frankwillemsi*; *C. franswittei*; *C. georgesmertensi*; *C. gillardinae*; *C. gistelincki*; *C. glacicremoratus*; *C. haplochromii* Paperna & Thurston, 1969; *C. irenae*; *C. jeanloujustinei* Rahmouni, Vanhove & Šimková, 2017; *C. longipenis* Paperna & Thurston, 1969; *C. makasai*; *C. milangelnari* Rahmouni, Vanhove & Šimková, 2017; *C. mulimbwai*; *C. muterezii*; *C. nageus* Řehulková, Mendlová & Šimková, 2013; *C. raeymaekersi*; *C. rognoni* Pariselle, Bilong Bilong & Euzet, 2003; *C. schreyenbrichardorum*; *C. sanjeani* Pariselle & Euzet, 1997; *C. sigmocirrus* Pariselle, Bitja Nyom & Bilong Bilong, 2014; *C. steenbergei*; *C. tilapiae* Paperna, 1960; *C. vandekerkhovei*; and *C. vealli*. Dorsal and ventral bars as well as the accessory piece in *C. adkoningsi* sp. nov. and *C. sturmbaueri* described from *O. ventralis* and *O. nasuta* are of similar size. Additionally, the copulatory duct in both species is similarly shaped (see Vanhove, Volckaert & Pariselle, 2011). However, the new species is distinguishable from *C. sturmbaueri* by (i) the longer dorsal anchors (30–32 μm in *C. adkoningsi* sp. nov. vs 19–21 μm in *C. sturmbaueri*), (ii) the longer dorsal bar auricles (35–44 μm in *C. adkoningsi* sp. nov. vs 12–15 μm in *C. sturmbaueri*), (iii) the different hook pairs (*C. adkoningsi* sp. nov. exhibits short hook pairs I–IV, VI, and VII while *C. sturmbaueri* displays short hook pair I and long pairs II–IV, VI, and VII (see Pariselle & Euzet, 2009; Rahmouni, Vanhove & Šimková, 2017 for the importance of hook length in the systematics of *Cichlidogyrus*), and (iv) the accessory piece (C-shaped, thick in the middle part ending in hook in *C. adkoningsi* sp. nov. vs H-shaped in *C. sturmbaueri*). The long auricles of *C. adkoningsi* sp. nov. are reminiscent of *C. vandekerkhovei* from *O. ventralis*, *O. boops* and *O. nasuta*, *C. glacicremoratus* from *O. nasuta*, and *C. discophonum* from *A. dewindti*, which are all parasites of ectodine cichlids (Vanhove, Volckaert & Pariselle, 2011; Rahmouni, Vanhove & Šimková, 2017). Further, *C. adkoningsi* sp. nov. shares with *C. makasai* similarly sized dorsal and ventral bars, hook pairs, and accessory pieces. However, in *C. adkoningsi* sp. nov. (i), the dorsal anchors are longer (30–32 μm in *C. adkoningsi* sp. nov. vs 19–23 μm in *C. makasai*), (ii) the dorsal bar auricles are much longer (35–44 in *C. adkoningsi* sp. nov. vs 17–23 μm in *C. makasai*), (iii) the heel is longer (9–11 in *C. adkoningsi* sp. nov. vs 2–4 μm in *C. makasai*), (iv) the copulatory duct is much shorter and differently shaped (relatively short copulatory duct, slightly curved halfway, with a length of 33–29 μm in *C. adkoningsi* sp. nov. vs thin curved duct which tapers distally, 69–79 μm long in *C. makasai*), and (v) the accessory piece is differently shaped (thick, C-shaped, ending in a hook in *C. adkoningsi* sp. nov. vs simple, slightly bent at distal third, resembling a spanner in *C. makasai*).

**Type-host:** *Cyphotilapia frontosa* (Boulenger, 1906) (Perciformes Bleeker, 1859: Cichlidae Heckel, 1840: Cyphotilapiini Salzburger et al., 2002).

**Host accession numbers: MH297995–96**

**Site of infection:** Gills.

**Type-locality:** Makabola village (3°32′S, 29°9′E; purchase from fisherman), DRC, LT.

**Prevalence & intensity of infection:** one fish specimen infected/two fish specimens examined, three parasite specimens on the infected fish.

**Holotype**: MRAC M.T.38432.

**Paratype**: MRAC M.T.38433.

**Etymology:** the specific epithet of the new species, ‘*adkoningsi*’, honours the Dutch biologist Dr. Adrianus Johannes Franciscus Marinus Maria Konings, known as Ad Konings, who has published extensively on cichlids. His books on the cichlids of LT have been crucial to our research on the parasite fauna of these fishes.

**Figure 4:**
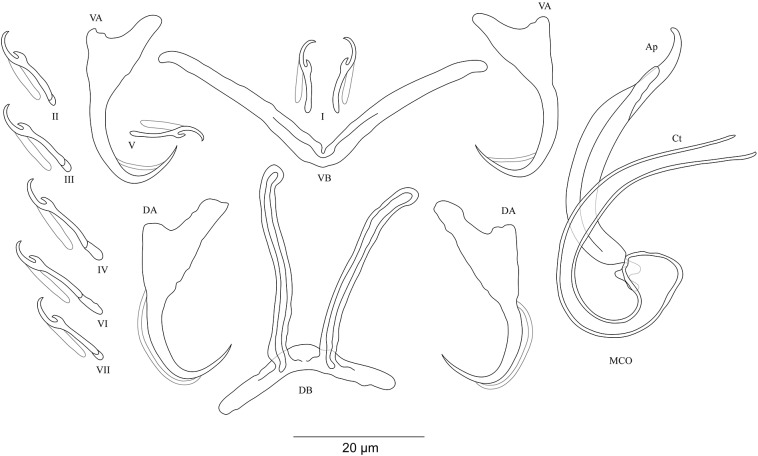
Sclerotized structures of *Cichlidogyrus koblmuelleri* sp. nov. ex *Cardiopharynx schoutedeni.* DA, dorsal anchor; DB, dorsal bar; VA, ventral anchor; VB, ventral bar; I–VII, hooks; MCO, male copulatory organ; Ct, copulatory tube; Ap, accessory piece.

***Cichlidogyrus koblmuelleri* sp. nov. Fig. 4**
urn:lsid:zoobank.org:act:473DB764-6798-43EE-8DD8-3B10D1AC1BBF.

**Description.** Based on seven specimens fixed in GAP. Body 462 (402–564; *n* = 4) long, 85 (68–121; *n* = 4) wide at mid-body. Dorsal anchors long with short shaft and more pronounced guard, curved blade and arched point: *a* = 27 (26–28; *n* = 4); *b* = 22 (21–23; *n* = 4); *c* = 1–2 (*n* = 4); *d* = 9 (8–10; *n* = 4); *e* = 9–10 (*n* = 4). Dorsal bar slightly curved with blunt endings and long auricles: *h* = 26 (25–28; *n* = 4); *w* = 4–5 (*n* = 4); *x* = 26 (25–27; *n* = 4); *y* = 4 (2–5; *n* = 4). Ventral anchors similar to dorsal ones: *a* = 24–25 (*n* = 4); *b* = 21–22 (*n* = 4); *c* = 1–2 (*n* = 4), *d* = 6 (5–7; *n* = 4); *e* = 9–10 (*n* = 4). V-shaped ventral bar: *w* = 2–3 (*n* = 4); *x* = 29 (28–30; *n* = 10). Haptor with seven short hook pairs, hooks V retain their larval size (see above), each hook with erect thumb and shank comprised of two subunits: pair I = 11 (10–12; *n* = 4) long, pair II = 13–14 (*n* = 4) long, pair III = 14–15 (*n* = 4) long, pair IV = 15–16 (*n* = 4) long, pair V = 10–11 (*n* = 4) long, pair VI = 16 (15–17; *n* = 4) long, and pair VII = 13–14 (*n* = 4) long. Male copulatory organ with a C-shaped copulatory duct: MCO = 46 (36–49; *n* = 7); Ct = 63 (59–65; *n* = 7). No heel. Accessory piece curved with two superimposed parts, one of them thicker and longer than the other, ending in moderately curved hook, Ap = 38 (37–40; *n* = 7). Vagina non-sclerotized.

**Diagnosis.** *Cichlidogyrus koblmuelleri* sp. nov. belongs to the same group as *C. adkoningsi* sp. nov. as it shares the small size of all hook pairs. The new species most closely resembles *C. discophonum* described from *A. dewindti* (see Rahmouni, Vanhove & Šimková, 2017) regarding the morphology of the dorsal and ventral anchors and the absence of a heel. However, it differs from the latter by (i) the longer copulatory duct (59–65 μm in *C. koblmuelleri* sp. nov. vs 41–47 μm in *C. discophonum*), and (ii) the longer and differently shaped accessory piece (curved with two superimposed parts, one of them thicker and longer than the other ending in a hook, 37–40 μm in *C. koblmuelleri* sp. nov. vs short with two thick distinct parts, twisted distally, ending in hook, 15–22 μm in *C. discophonum*). Like *C. adkoningsi* sp. nov. and the other *Cichlidogyrus* spp. of the ectodine cichlids listed in the previous diagnosis, *C. koblmuelleri* sp. nov. exhibits long dorsal bar auricles. However, they are longer in the new species than those of *C. glacicremoratus* (25–28 μm in *C. koblmuelleri* sp. nov. vs 14–18 μm in *C. glacicremoratus*). Moreover, *C. koblmuelleri* sp. nov. is mainly distinguishable from *C. glacicremoratus* by (i) the longer and differently shaped copulatory duct (C-shaped copulatory duct, 59–65 μm in *C. koblmuelleri* sp. nov. vs wavy copulatory duct, with thick wall, constricted and curved approximately at proximal third, with wide terminal opening, 42–47 μm in *C. glacicremoratus*), and (ii) the heel (no heel in *C. koblmuelleri* sp. nov. vs small, irregular sclerotized flange-like structure in *C. glacicremoratus*).

**Type-host:** *Cardiopharynx schoutedeni* Poll, 1942 (Perciformes Bleeker, 1859: Cichlidae Heckel, 1840: Ectodini Poll, 1986).

**Host accession numbers: MH297989–94.**

**Site of infection:** Gills.

**Type-locality:** Mulongwe fish market (3°22′S, 29°6′E), Uvira, DRC, LT.

**Prevalence & intensity of infection:** four fish specimens infected/six fish specimens examined, one to three parasite specimens per infected host.

**Holotype**: MRAC M.T.38434.

**Paratypes**: MRAC M.T.38435, MRAC M.T.38439.

**Etymology:** the specific epithet of the new species, ‘*koblmuelleri*’ honours the biologist Dr. Stephan Koblmüller (Austria), an all-round specialist in LT’s ichthyofauna, in recognition of his crucial contribution to parasitological work.

**Figure 5:**
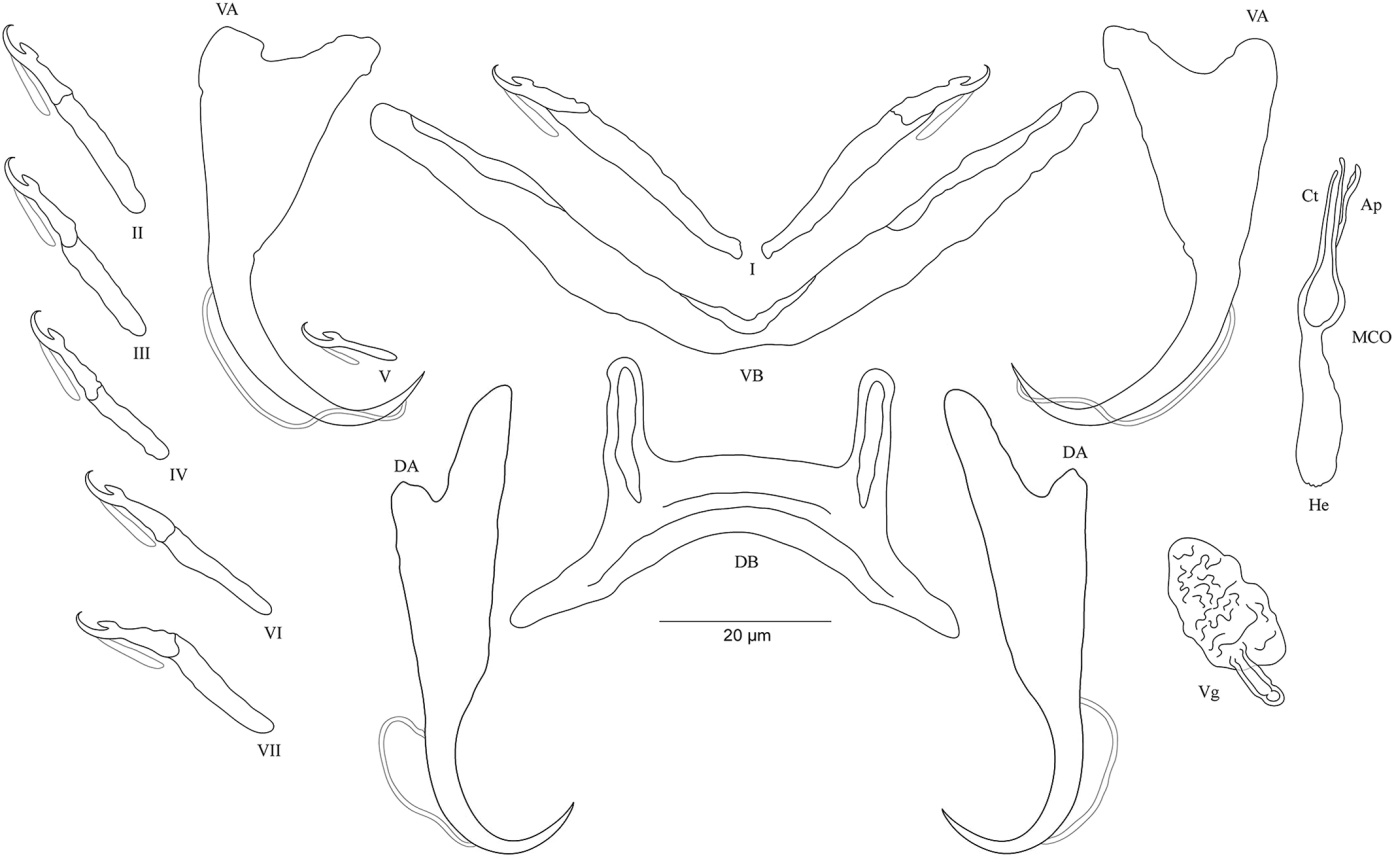
Sclerotized structures of *Cichlidogyrus habluetzeli* sp. nov. ex *Cardiopharynx schoutedeni* and *Cyphotilapia frontosa.* DA, dorsal anchor; DB, dorsal bar; VA, ventral anchor; VB, ventral bar; I–VII, hooks; MCO, male copulatory organ; He, heel; Ct, copulatory tube; Ap, accessory piece; Vg, vagina.

***Cichlidogyrus habluetzeli* sp. nov. Fig. 5**
urn:lsid:zoobank.org:act:EC79CE69-2D88-4D96-A255-AC0E31B76EAE.

**Description.** Based on 28 specimens fixed in GAP. Body 507 (286–790; *n* = 19) long, 99 (62–152; *n* = 19) wide at mid-body. Dorsal anchors long with short shaft and more pronounced guard, curved blade with slightly arched point: *a* = 60 (53–66; *n* = 20); *b* = 43 (38–46; *n* = 20); *c* = 6 (3–9; *n* = 21); *d* = 22 (16–26; *n* = 20); *e* = 11 (9–14; *n* = 20). Dorsal bar relatively curved, thick in the middle part with blunt endings and straight auricles: *h* = 14 (10–18; *n* = 19); *w* = 11 (9–14; *n* = 18); *x* = 59 (54–63; *n* = 18); *y* = 25 (22–29; *n* = 19). Ventral anchors shorter than dorsal ones with shorter shaft than guard, curved blade with arched point: *a* = 47 (44–52; *n* = 12); *b* = 44 (40–48; *n* = 20); *c* = 6 (5–8; *n* = 18), *d* = 12 (8–15; *n* = 18); *e* = 11 (10–13; *n* = 21). Long and thick V-shaped ventral bar: *w* = 7 (6–10; *n* = 20); *x* = 53 (47–60; *n* = 20). Haptor with seven long hook pairs, pair I large in comparison with remaining pairs, hooks V retain their larval size (*sensu* Pariselle & Euzet, 2003, 2009), and each hook with erect thumb and shank comprised of two subunits: pair I = 38 (33–42; *n* = 20) long, pair II = 27 (23–31; *n* = 18) long, pair III = 27 (22–31; *n* = 19) long, pair IV = 24 (21–28; *n* = 19) long, pair V = 12 (11–13; *n* = 19) long, pair VI = 28 (24–31; *n* = 20) long, and pair VII = 28 (25–32; *n* = 20) long. Male copulatory organ with a relatively short straight copulatory duct: MCO = 44 (39–53; *n* = 23); Ct = 21 (19–23; *n* = 24). Well-developed straight heel, He = 23 (18–29; *n* = 24). Thin accessory piece distally with leaf-shaped ending, Ap = 15 (13–21; *n* = 21). Sclerotized vagina: V = 23 (19–32; *n* = 6); v = 11 (10–14; *n* = 7).

**Diagnosis.** *Cichlidogyrus habluetzeli* sp. nov. belongs to the group of species with long hook pairs I–IV, VI and VII, large hook pair I, a copulatory duct without a swollen proximal portion, and a sclerotized vagina (see above). This group is restricted to a single species, the Tanganyikan *C. centesimus* (see Vanhove, Volckaert & Pariselle, 2011). Therefore, *C. habluetzeli* sp. nov. is only the second record with this configuration of sclerotized structures throughout the entire genus. The dorsal bar auricles in *C. habluetzeli* sp. nov. are small hollow outgrowths on the anterior face, a feature observed in congeners infecting representatives of Tylochromini and Ectodini. With this morphology, we find Tanganyikan *C. mulimbwai* and *C. muzumanii*, both from *T. polylepis* (Muterezi Bukinga et al., 2012), *C. pseudoaspiralis* from *A. dewindti*, and *C. aspiralis* from *O. nasuta* (Rahmouni, Vanhove & Šimková, 2017) to be just like non-Tanganyikan *C. chrysopiformis*, *C. djietoi*, and *C. sigmocirrus* Pariselle, Bitja Nyom & Bilong Bilong, 2014, all parasites of *T. sudanensis* Daget, 1954 (Pariselle, Bitja Nyom & Bilong Bilong, 2014), *C. kothiasi* Pariselle & Euzet, 1994 from *T. jentinki* (Steindachner, 1862) (Pariselle & Euzet, 1994), and *C. dageti*, *C. euzeti*, and *C. falcifer* Dossou & Birgi, 1984 from *Hemichromis fasciatus* (Dossou & Birgi, 1984; Pariselle & Euzet, 2009). In addition to its haptoral features, *C. habluetzeli* sp. nov. exhibits a similar morphotype of the reproductive organs as *C. aspiralis*, *C. pseudoaspiralis* (Rahmouni, Vanhove & Šimková, 2017), *C. casuarinus* Pariselle, Muterezi Bukinga & Vanhove, 2015 isolated from a range of cichlid representatives of the tribe Bathybatini Poll, 1986 (Pariselle et al., 2015a), *C. centesimus* (Vanhove, Volckaert & Pariselle, 2011), and *C. nshomboi* Muterezi Bukinga et al., 2012 from *Boulengerochromis microlepis* (Boulenger, 1899) Boulengerochromini Takahashi, 2003 (all Tanganyikan species) (Muterezi Bukinga et al., 2012). *C. habluetzeli* sp. nov. can be compared to *C. aspiralis* regarding the similarly shaped and sized copulatory duct and accessory piece, and the presence of a sclerotized vagina. However, the new species differs from *C. aspiralis* by (i) the longer dorsal and ventral anchors (respectively, 60–66; 46–52 μm in *C. habluetzeli* sp. nov. vs 39–43; 33–34 μm in *C. aspiralis*), (ii) the longer dorsal and ventral bars (respectively, 54–63; 52–60 μm in *C. habluetzeli* sp. nov. vs 45–47, 37–40 μm in *C. aspiralis*), (iii) and the longer dorsal bar auricles (14–18 μm in *C. habluetzeli* sp. nov. vs 6–10 μm in *C. aspiralis*). On the other hand, the dorsal and ventral anchors in *C. habluetzeli* sp. nov. are similar to those of *C. casuarinus* and *C. nshomboi*, while in *C. centesimus* and *C. pseudoaspiralis*, they are shorter (respectively, 53–66; 44–52 μm in *C. habluetzeli* sp. nov. vs 41–55; 34–44 μm in *C. centesimus* and 37–42; 37–40 μm in *C. pseudoaspiralis*). Also, the dorsal bar in the new species is similar to that of *C. nshomboi*, shorter than that of *C. casuarinus* (54–63 μm in *C. habluetzeli* sp. nov. vs 64–85 μm in *C. casuarinus*), and longer than those of *C. centesimus* and *C. pseudoaspiralis* (54–63 μm in *C. habluetzeli* sp. nov. vs 37–52; 31–32 μm in *C. centesimus*, and *C. pseudoaspiralis*, respectively). Like *C. aspiralis* and *C. pseudoaspiralis*, *C. habluetzeli* sp. nov. lacks a spirally coiled thickening in the distal part of its copulatory duct, a feature present in *C. casuarinus, C. centesimus* and *C. nshomboi*. Further, the copulatory duct in *C. habluetzeli* sp. nov. is similar in size to that in *C. centesimus* and *C. nshomboi*, while it is longer in *C. casuarinus and C. pseudoaspiralis* (19–23 μm in *C. habluetzeli* sp. nov. vs 33–44; 40–43 μm in *C. casuarinus and C. pseudoaspiralis*, respectively). Unlike *C. pseudoaspiralis*, *C. habluetzeli* sp. nov. exhibits a sclerotized vagina similarly to *C. casuarinus*, *C. centesimus*, and *C. nshomboi* (see Vanhove, Volckaert & Pariselle, 2011; Muterezi Bukinga et al., 2012; Pariselle et al., 2015a; Rahmouni, Vanhove & Šimková, 2017).

**Type-host:** *Cardiopharynx schoutedeni* Poll, 1942 (Perciformes Bleeker, 1859: Cichlidae Heckel, 1840: Ectodini Poll, 1986).

**Site of infection:** Gills.

**Type-locality:** Mulongwe fish market (3°22′S, 29°6′E), Uvira, DRC, LT.

**Prevalence & intensity of infection in the type host:** three fish specimens infected/six fish specimens examined, one to six parasite specimens per infected host.

**Additional host**: found on the gills of *C. frontosa*, one fish specimen infected/two fish specimens examined, nine parasite specimens on the infected host.

**Holotype**: MRAC M.T.38437.

**Paratypes**: MRAC M.T.38436, MRAC M.T.38438; MNHN HEL748–49, HEL752; MZH KN10058–59.

**Etymology:** the specific epithet of the new species, ‘*habluetzeli*’ honours the biologist Dr. Pascal István Hablützel (Switzerland/Belgium) in honour of his pioneering work in the eco-immunology of Tanganyikan cichlids.

**Figure 6:**
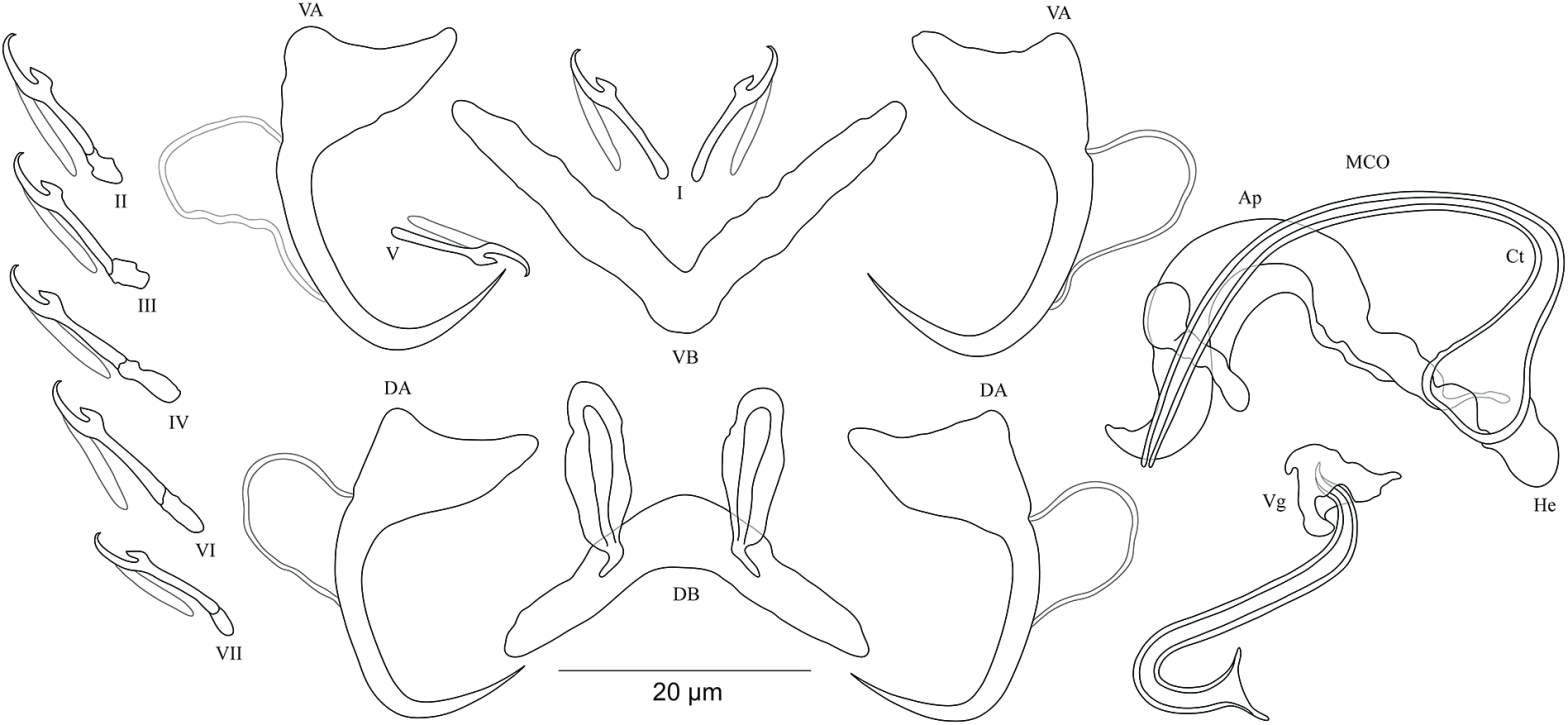
Sclerotized structures of *Cichlidogyrus antoineparisellei* sp. nov. ex *Interochromis loocki.* DA, dorsal anchor; DB, dorsal bar; VA, ventral anchor; VB, ventral bar; I–VII, hooks; MCO, male copulatory organ; He, heel; Ct, copulatory tube; Ap, accessory piece; Vg, vagina.

***Cichlidogyrus antoineparisellei* sp. nov. Fig. 6**
urn:lsid:zoobank.org:act:B324AD44-4520-4129-8DBB-8985A981783D.

**Description.** Based on 12 specimens fixed in GAP. Body 436 (330–509; *n* = 5) long, 82 (76–86; *n* = 5) wide at mid-body. Dorsal anchors with poorly marked shaft and more pronounced guard (approximately four times the length of shaft), bent blade with arched point: *a* = 25 (24–26; *n* = 7); *b* = 22–23 (*n* = 7); *c* = 1–2 (*n* = 7); *d* = 6 (4–7; *n* = 7); *e* = 9 (8–10; *n* = 7). Dorsal bar curved, thick in the middle part with blunt endings and well-developed auricles: *h* = 11–12 (*n* = 7); *w* = 5–6 (*n* = 7); *x* = 27 (26–29; *n* = 7); *y* = 9 (8–11; *n* = 7). Ventral anchors similar in shape and size to dorsal ones: *a* = 26 (25–27; *n* = 7); *b* = 24 (23–25; *n* = 7); *c* = 2 (1–3; *n* = 7); *d* = 6 (3–7; *n* = 7); *e* = 10 (9–12; *n* = 7). V-shaped ventral bar: *w* = 4–5 (*n* = 7); *x* = 26 (24–27; *n* = 7). Haptor with seven short hook pairs, hooks V retain their larval size (see above), each hook with erect thumb and shank comprised of two subunits: pair I = 11–12 (*n* = 6) long, pair II = 13 (12–14; *n* = 7) long, pair III = 15 (13–16; *n* = 7) long, pair IV = 17 (15–18; *n* = 7) long, pair V = 11 (9–12; *n* = 6) long, pair VI = 16 (14–17; *n* = 7) long, and pair VII = 13–14 (*n* = 7) long. Male copulatory organ composed of long, curved copulatory duct that tapers distally: MCO = 42 (39–46; *n* = 10); Ct = 59 (54–62; *n* = 11). Heel relatively short, He = 6 (5–7; *n* = 11). Accessory piece is linked to basal bulb by thin filament, C-shaped in the middle part, composed of two distinct parts, one extremity ending in hook while the other connected to the latter with swollen base, Ap = 29 (22–33; *n* = 10). Long tubular vagina with two characteristic extremities, a broadened part and one extremity covered by a triangular-like structure: V = 40 (35–47; *n* = 11); *v* = 9 (7–13; *n* = 11).

**Diagnosis.** According to the sclerites of the haptoral and reproductive organs, *C. antoineparisellei* sp. nov. belongs to the group which includes species with short hook pairs I–IV, VI, and VII, and a copulatory duct without a swollen proximal portion, and exhibits a sclerotized vagina (see above). This group includes *C. acerbus* Dossou, 1982; *C. amieti* Birgi & Euzet, 1983; *C. amphoratus* Pariselle & Euzet, 1996; *C. berrebii* Pariselle & Euzet, 1994; *C. cirratus* Paperna, 1964; *C. cubitus* Dossou, 1982; *C. djietoi*; *C. giostrai* Pariselle, Bilong Bilong & Euzet, 2003; *C. karibae* Douëllou, 1993; *C. kothiasi* Pariselle & Euzet, 1994; *C. lagoonaris* Paperna, 1969; *C. levequei* Pariselle & Euzet, 1996; *C. louipaysani* Pariselle & Euzet, 1995; *C. mvogoi* Pariselle, Bitja Nyom & Bilong Bilong, 2014; *C. njinei* Pariselle, Bilong Bilong & Euzet, 2003; *C. ornatus* Pariselle & Euzet, 1996; *C. pouyaudi* Pariselle & Euzet, 1994; *C. sclerosus* Paperna & Thurston, 1969; *C. slembroucki* Pariselle & Euzet, 1998; and *C. zambezensis Douëllou*, 1993. *C. mbirizei* Muterezi Bukinga et al., 2012, a parasite described from a representative of the tribe Oreochromini Dunz & Schliewen, 2013, *Oreochromis tanganicae* (Günther, 1894), is the only species hitherto known to have short hook pairs I–IV, VI, and VII and a sclerotized vagina in LT (see Muterezi Bukinga et al., 2012), and therefore *C. antoineparisellei* sp. nov. is the second known representative with the combination of these sclerotized structures in the lake. However, *C. antoineparisellei* sp. nov. is the first representative of *Cichlidogyrus* recognized on Tanganyikan tropheines which has a sclerotized vagina. Further, because of the systematic and phylogenetic affinities among the Tropheini and the Haplochromini Poll, 1986 (see Salzburger et al., 2005), *C. antoineparisellei* sp. nov. is considered to be the second representative species exhibiting this feature within the parasites infecting representatives of the Haplochromini lineage, like its congener *C. zambezensis* reported from the non-Tanganyikan haplochromines *Serranochromis macrocephalus* (Boulenger, 1899), *S. robustus jallae* (Günther, 1864) (Vanhove et al., 2013), *S. mellandi* (Boulenger, 1905), *S. stappersi* Trewavas, 1964, *S. thumbergi* (Castelnau, 1861), and *S. angusticeps* (Boulenger, 1905) (*C. zambezensis* was also found on *O. mortimeri* Trewavas, 1966; see Douëllou, 1993; Jorissen et al., 2018). Morphologically, *C. antoineparisellei* sp. nov. exhibits similar haptoral features to those of Tanganyikan *C. brunnensis* described from *Trematocara unimaculatum* Boulenger, 1901 Trematocarini Poll, 1986 (Kmentová et al., 2016a), and a variety of West African parasite species such as *C. amphoratus* from *Tilapia louka* Thys van den Audenaerde, 1969 (Pariselle & Euzet, 1996), *C. sclerosus* Paperna & Thurston, 1969 from *O. mossambicus* (Peters, 1852), *O. niloticus* (Linnaeus, 1758), *Haplochromis* sp., *O. leucosticus* (Trewavas, 1933), *C. zillii* (Gervais, 1848), *O. spilurus* (Günther, 1894), *O. aureus* (Steindachner, 1864) (Paperna & Thurston, 1969; Pariselle & Euzet, 2009), *O. mortimeri*, *S. microcephalus* (Douëllou, 1993), and *O. mweruensis* Trewavas, 1983 (Jorissen et al., 2018), and *C. giostrai* from *Sarotherodon caudomarginatus* (Boulenger, 1916) (Pariselle, Bilong Bilong & Euzet, 2003). These include the characteristic broad base and almost non-incised roots of the anchors. *C. antoineparisellei* sp. nov. shares its host species *I. loocki* with *C. buescheri* in addition to the morphology of its hook pairs and dorsal and ventral anchors (see Pariselle et al., 2015b). However, it is mainly distinguishable from the latter by (i) the shorter dorsal bar (26–29 μm in *C. antoineparisellei* sp. nov. vs 31–47 μm in *C. buescheri*), and (ii) the sclerotized vagina (no sclerotized vagina in *C. buescheri*).

**Type-host:** *Interochromis loocki* (Poll, 1949) (Perciformes Bleeker, 1859: Cichlidae Heckel, 1840: Tropheini Poll, 1986).

**Host accession numbers: MH298001–02.**

**Site of infection:** Gills.

**Type-locality:** Pemba (3°40′S, 29°10′E; caught when snorkelling), DRC, LT.

**Prevalence & intensity of infection:** two fish specimens infected/two fish specimens examined, one to seven parasite specimens per infected host.

**Holotype**: MRAC M.T.38440.

**Paratypes**: MRAC M.T.38441–43; MNHN HEL750–51.

**Etymology:** the species epithet ‘*antoineparisellei*’ honours the French parasitologist Dr. Antoine Pariselle, researcher at the Institut de Recherche pour le Développement, who extensively studied the monogeneans of African fish, trained countless people in fish parasitology, and described more than fifty species of *Cichlidogyrus*, of which three parasitize *I. loocki*.

**Figure 7:**
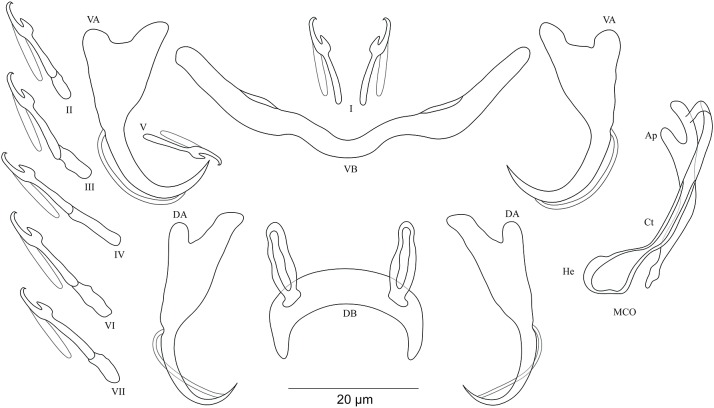
Sclerotized structures of *Cichlidogyrus masilyai* sp. nov. ex *Petrochromis orthognathus*. DA, dorsal anchor; DB, dorsal bar; VA, ventral anchor; VB, ventral bar; I–VII, hooks; MCO, male copulatory organ; He, heel; Ct, copulatory tube; Ap, accessory piece.

***Cichlidogyrus masilyai* sp. nov. Fig. 7**
urn:lsid:zoobank.org:act:4D5AF9E3-9CCE-4D3F-95B4-CCDA7B1A5C91.

**Description.** Based on 12 specimens fixed in GAP. Body 622 (523–721; *n* = 2) long, 107 (56–158; *n* = 2) wide at mid-body. Dorsal anchors with short shaft and pronounced guard (approximately two times the length of shaft), bent blade with arched point: *a* = 28 (27–29; *n* = 3); *b* = 21 (20–23; *n* = 3); *c* = 5 (4–6; *n* = 3); *d* = 10 (9–11; *n* = 3); *e* = 7 (5–8; *n* = 4). Dorsal bar well-arched, C-shaped with relatively short auricles: *h* = 10 (8–11; *n* = 3); *w* = 6 (5–7; *n* = 3); *x* = 28 (22–24; *n* = 3); *y* = 14 (13–15; *n* = 3). Ventral anchors with shorter shaft than guard and arched point: *a* = 28 (27–29; *n* = 3); *b* = 24 (23–25; *n* = 3); *c* = 4 (2–5; *n* = 3); *d* = 9 (6–10; *n* = 3); *e* = 9 (8–10; *n* = 3). W-shaped ventral bar: *w* = 5 (4–6; *n* = 3); *x* = 32 (31–33; *n* = 3). Haptor with seven short hook pairs, hooks V retain their larval size (see above), each hook with erect thumb and shank comprised of two subunits: pair I = 12–13 (*n* = 3) long, pair II = 16 (15–17; *n* = 3) long, pair III = 20 (19–21; *n* = 3) long, pair IV = 22 (21–23; *n* = 3) long, pair V = 12 (10–13; *n* = 3) long, pair VI = 22–23 (*n* = 3) long, and pair VII = 19 (17–21; *n* = 3) long. Male copulatory organ beginning as an irregularly shaped bulb with thin copulatory duct, slightly curved proximally, straight halfway, folded back distally: MCO = 33 (31–34; *n* = 3); Ct = 35 (32–36; *n* = 3). Poorly developed heel at the side of the basal bulb, He = 1–2 (*n* = 3). Accessory piece seems to be unattached to basal bulb, is thin, slightly curved in the middle part with pincer-like ending, Ap = 26 instead of 29 (24–29; *n* = 3). Vagina non-sclerotized.

**Diagnosis.** *Cichlidogyrus masilyai* sp. nov. belongs to the same group as *C. adkoningsi* sp. nov. and *C. koblmuelleri* sp. nov. (see above). The new species shows a similar morphotype to several representatives of *Cichlidogyrus* described from non-Tanganyikan haplochromine cichlids such as *C. bifurcatus* from *Astatotilapia flaviijosephi* (Lortet, 1883) (Paperna, 1960), *C. haplochromii* from *H. guiarti* (Pellegrin, 1904) (Paperna & Thurston, 1969) and *C. zambezensis*, in addition to *C. gillardinae* from the Tanganyikan haplochromine *A. burtoni* (Günther, 1894) (Muterezi Bukinga et al., 2012). Further, the exceptional shape of the dorsal bar in the new species, that is, well-arched, C-shaped, is observed for the first time in *Cichlidogyrus* spp. infecting a tropheine cichlid. The new species exhibits the same hook pairs, similarly sized dorsal and ventral anchors, a non-sclerotized vagina, and an accessory piece ending in a pincer-like structure as in *C. buescheri*, a parasite of *I. loocki*. However, the shape of the dorsal and ventral anchors is different, that is, the shaft is more clearly marked in *C. masilyai* sp. nov. compared to that observed in *C. buescheri*. Further, it differs from *C. buescheri* by (i) the shorter and differently shaped dorsal bar (C-shaped, 22–24 μm in *C. masilyai* sp. nov. vs moderately arched, 31–45 μm in *C. buescheri*), (ii) the copulatory duct (thin, slightly curved proximally, straight halfway, folded distally, 31–34 μm in *C. masilyai* sp. nov. vs curved duct with narrow extremity, 49–58 μm in *C. buescheri*), (iv) the heel (poorly developed, lateral to the bulb, 1–2 μm in *C. masilyai* sp. nov. vs large heel of irregular shape at the basis of the bulb, 6–11 μm in *C. buescheri*), and (v) the accessory piece (seems to be unattached to basal bulb and thin, 24–29 μm in *C. masilyai* sp. nov. vs wide, directly attached to the basal bulb, 31–52 μm in *C. buescheri*).

**Type-host:** *Petrochromis orthognathus* Matthes, 1959 (Perciformes Bleeker, 1859: Cichlidae Heckel, 1840: Tropheini Poll, 1986).

**Host accession numbers: MH298003–06.**

**Site of infection:** Gills.

**Type-locality:** Pemba (3°40′S, 29°10′E; caught when snorkelling), DRC, LT.

**Prevalence & intensity of infection:** two fish specimens infected/four fish specimens examined, one to two parasite specimens per infected host.

**Holotype**: MRAC M.T.38447.

**Paratype**: MRAC M.T.38446.

**Etymology:** the species epithet ‘*masilyai*’ is given in honour of the Congolese biologist Prof. Dr. Pascal Masilya Mulungula of the Institut Supérieur Pédagogique de Bukavu and general director of the Centre de Recherche en Hydrobiologie-Uvira (DRC), in appreciation of the hospitality and help received during the fieldtrip for the present study.

**Figure 8:**
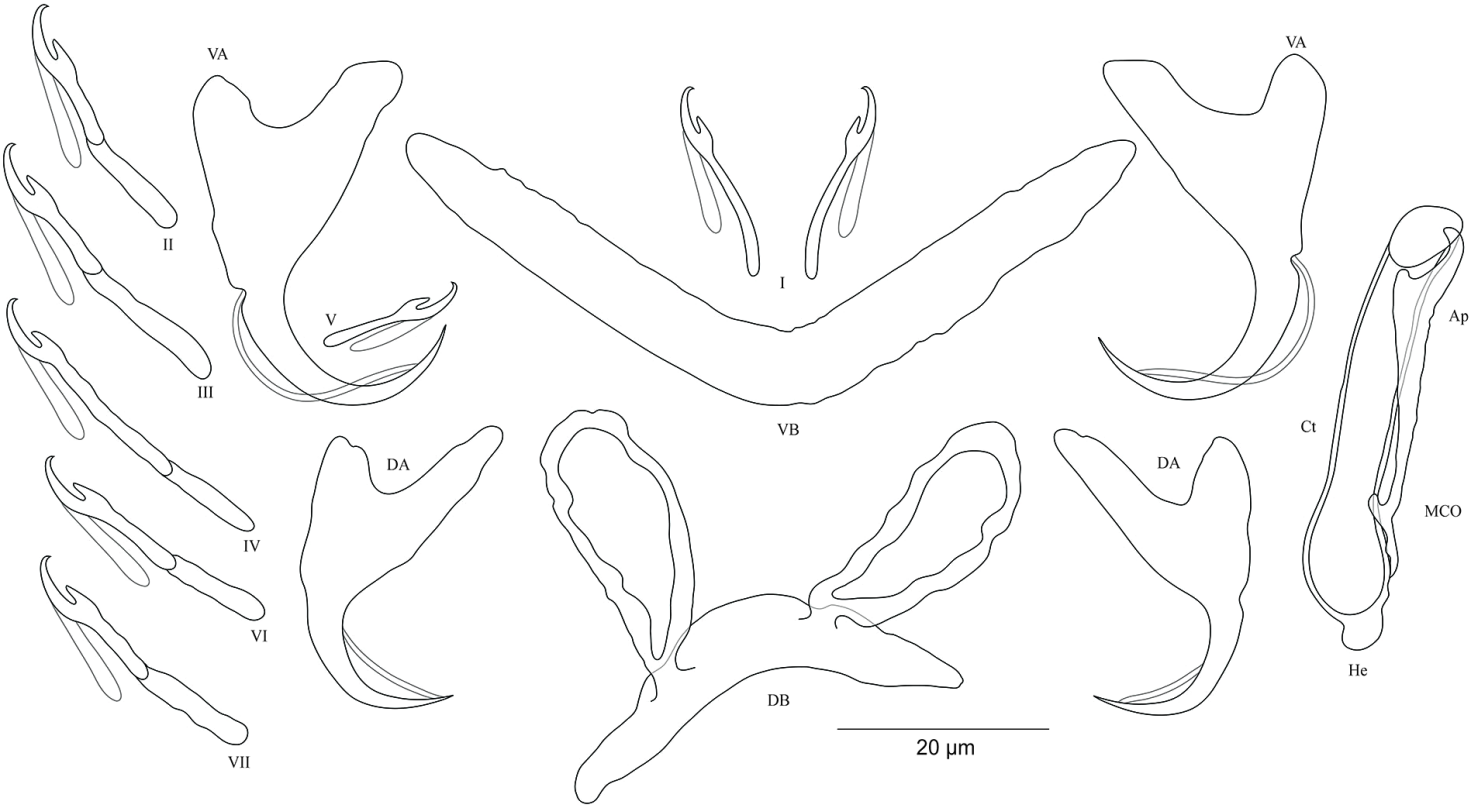
Sclerotized structures of *Cichlidogyrus salzburgeri* sp. nov. ex *Petrochromis trawavasae*. DA, dorsal anchor; DB, dorsal bar; VA, ventral anchor; VB, ventral bar; I–VII, hooks; MCO, male copulatory organ; He, heel; Ct, copulatory tube; Ap, accessory piece.

***Cichlidogyrus salzburgeri* sp. nov. Fig. 8**
urn:lsid:zoobank.org:act:612A6D2B-09DD-4902-8640-A4877277E7F3.

**Description.** Based on four specimens fixed in GAP. Dorsal anchors with short shaft and more pronounced guard (approximately three times the length of shaft), bent blade with arched point: *a* = 27 (26–29; *n* = 3); *b* = 18 (17–19; *n* = 3); *c* = 4–5 (*n* = 3); *d* = 12–13 (*n* = 3); *e* = 8 (7–9; *n* = 4). Dorsal bar slightly curved with well-developed auricles: *h* = 22 (21–24; *n* = 3); *w* = 6–7 (*n* = 3); *x* = 32 (35–38; *n* = 3); *y* = 11–12 (*n* = 4). Ventral anchors with shorter shaft than guard and arched point: *a* = 32 (31–33; *n* = 3); *b* = 26–27 (*n* = 3); *c* = 5–6 (*n* = 3); *d* = 11–12 (*n* = 3); *e* = 12–13 (*n* = 3). V-shaped ventral bar: *w* = 7–8 (*n* = 3); *x* = 39 (38–40; *n* = 3). Haptor with short hook pair I, hooks V retain their larval size (see above), pairs II–IV, VI, and VII long, each hook with erect thumb and shank comprised of two subunits: pair I = 12–13 (*n* = 3) long, pair II = 23–24 (*n* = 3) long, pair III = 28–29 (*n* = 3) long, pair IV = 31–32 (*n* = 3) long, pair V = 11–12 (*n* = 3) long, pair VI = 25–26 (*n* = 3) long, and pair VII = 25–26 (*n* = 3) long. Male copulatory organ with straight, wide copulatory duct and large distal opening: MCO = 42 (41–44; *n* = 4); Ct = 40 (39–42; *n* = 4). Short heel, He = 3 (2–4; *n* = 4). Accessory piece linked to basal bulb, thin proximally, straight, pincer-like ending with one extremity shorter than the other, Ap = 29 (28–30; *n* = 4). Vagina non-sclerotized.

**Diagnosis.** *Cichlidogyrus salzburgeri* sp. nov. belongs to the group of *Cichlidogyrus* which are characterized by a shorter hook pair I (pair V with larval size) and longer pairs II–IV, VI, and VII, a copulatory duct without a swollen distal portion, and a non-sclerotized vagina. Of all *Cichlidogyrus* spp., only *C. halli* Price & Kirk, 1967 and the Tanganyikan *C. sturmbaueri* (Vanhove, Volckaert & Pariselle, 2011) and *C. rectangulus* (Rahmouni, Vanhove & Šimková, 2017) are included in this group. Therefore, the new species is the third representative described from endemic Tanganyikan cichlids displaying this hook configuration. *C. salzburgeri* sp. nov. differs from its congeners *C. buescheri*, *C. schreyenbrichardorum*, and *C. vealli* isolated from *I. loocki*, and from the undescribed species previously identified on some representatives of *Petrochromis*, by the shape of the anchors, specifically the marked bases in the ventral ones (Pariselle et al., 2015b). The dorsal bar in *C. salzburgeri* sp. nov. with its well-developed auricles is reminiscent of *C. muterezii* described from *S. diagramma* (see Van Steenberge et al., 2015). However, *C. salzburgeri* sp. nov. differs from the latter by (i) the hook pairs (shorter hook pair I and longer pairs II–IV, VI, and VII in *C. salzburgeri* sp. nov. vs short hook pairs I–IV, VI, and VII in *C. muterezii*) and (ii) the pincer-like ending in the accessory piece, a feature missing in *C. muterezii*. Further, according to the comparative morphology of the reproductive organs, *C. salzburgeri* sp. nov. differs from *C. franswittei* found on the gills of *P. marginatus* and *P. curvifrons* (see Van Steenberge et al., 2015) by (i) the hook pairs (shorter hook pair I and longer pairs II–IV, VI, and VII in *C. salzburgeri* sp. nov. vs short hook pairs I–IV, VI, and VII in *C. franswittei*), (ii) the shorter dorsal anchors (26–29 μm in *C. salzburgeri* sp. nov. vs 31–40 μm in *C. franswittei*), (iii) the shorter copulatory duct (39–42 μm in *C. salzburgeri* sp. nov. vs 47–57 μm in *C. franswittei*), and (iv) the shorter accessory piece (28–30 μm in *C. salzburgeri* sp. nov. vs 31–46 μm in *C. franswittei*). *C. salzburgeri* sp. nov. is distinguishable from the newly described species *C. masilyai* sp. nov. by (i) the longer dorsal bar (35–38 μm in *C. salzburgeri* sp. nov. vs 22–24 μm in *C. masilyai* sp. nov.), (ii) the length of its auricles (21–24 μm in *C. salzburgeri* sp. nov. vs 8–11 μm in *C. masilyai* sp. nov.), (iii) the differently shaped copulatory duct (straight, large copulatory duct and large distal opening in *C. salzburgeri* sp. nov. vs thin, slightly curved proximally, straight halfway, folded distally in *C. masilyai* sp. nov.), and (v) the differently shaped accessory piece (linked to basal bulb, thin proximally, straight, ending pincer-like with one extremity shorter than the other in *C. salzburgeri* sp. nov. vs thin and slightly curved in the middle part ending in pincer-like structure in *C. masilyai* sp. nov.).

**Type-host:** *Petrochromis trewavasae* Poll, 1948 (Perciformes Bleeker, 1859: Cichlidae Heckel, 1840: Tropheini Poll, 1986).

**Host accession number: MH298007.**

**Site of infection:** Gills.

**Type-locality:** Pemba (3°40′S, 29°10′E; caught when snorkelling), DRC, LT.

**Prevalence & intensity of infection:** one fish specimen infected/two fish specimen examined, four parasite specimens on infected fish.

**Holotype**: MRAC M.T.38448.

**Paratype**: MRAC M.T.38449.

**Etymology:** the specific epithet ‘*salzburgeri*’ honours the biologist Dr. Walter Salzburger, professor at the University of Basel (Switzerland), for his work on cichlid evolution and in appreciation of his assistance during the fieldtrip.

**Figure 9:**
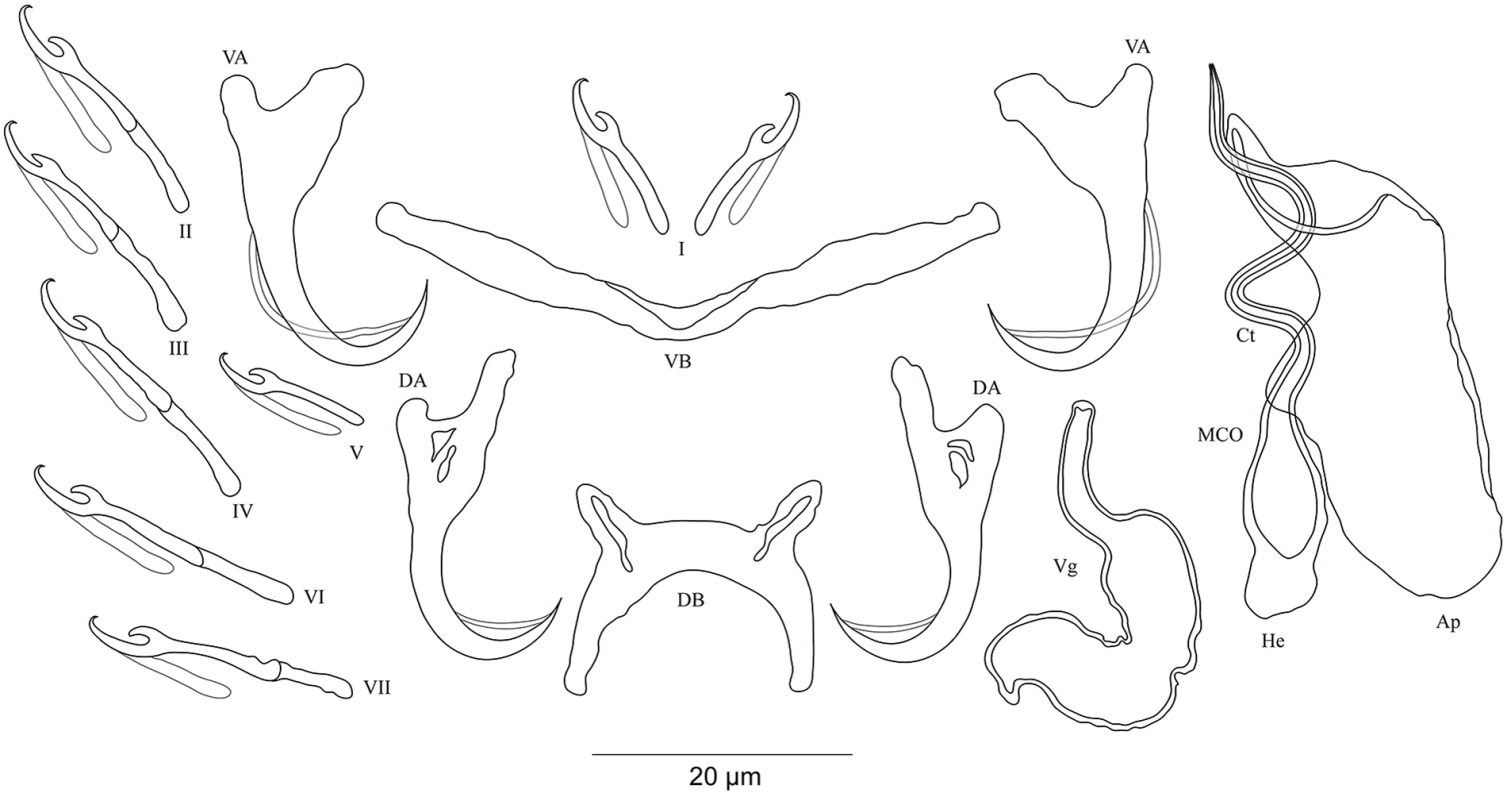
Sclerotized structures of *Cichlidogyrus sergemorandi* sp. nov. ex *Tylochromis polylepis*. DA, dorsal anchor; DB, dorsal bar; VA, ventral anchor; VB, ventral bar; I–VII, hooks; MCO, male copulatory organ; He, heel; Ct, copulatory tube; Ap, accessory piece; Vg, vagina.

***Cichlidogyrus sergemorandi* sp. nov. Fig. 9**
urn:lsid:zoobank.org:act:7A4653B3-2C25-4E3A-9D4A-3FA76165C167.

**Description.** Based on seven specimens fixed in GAP. Body 659 (594–769; *n* = 4) long, 115 (95–146; *n* = 4) wide at mid-body. Dorsal anchors with short shaft and more pronounced guard (approximately two times the length of shaft), slightly bent blade with arched point: *a* = 24 (23–26; *n* = 5); *b* = 19 (18–20; *n* = 5); *c* = 2–3 (*n* = 5); *d* = 7 (6–8; *n* = 5); *e* = 6–7 (*n* = 5). C-shaped dorsal bar with blunt endings and short auricles: *h* = 4–5 (*n* = 5); *w* = 3–4 (*n* = 5); *x* = 18 (16–20; *n* = 5); *y* = 9 (7–10; *n* = 5). Ventral anchors with shorter shaft than guard and arched point: *a* = 23 (22–24; *n* = 4); *b* = 20–21 (*n* = 5); *c* = 3 (2–4; *n* = 5), *d* = 7 (6–8; *n* = 5); *e* = 9 (8–10; *n* = 5). V-shaped ventral bar: *w* = 4–5 (*n* = 5); *x* = 26 (25–27; *n* = 5). Haptor with seven short hook pairs, hooks V retain their larval size, each hook with erect thumb and shank comprised of two subunits (see above): pair I = 13–14 (*n* = 5) long, pair II = 18 (17–19; *n* = 5) long, pair III = 21 (19–22; *n* = 5) long, pair IV = 22 (21–23; *n* = 5) long, pair V = 10 (9–11; *n* = 5) long, pair VI = 22 (21–24; *n* = 5) long, and pair VII = 22 (20–24; *n* = 4) long. Male copulatory organ with an elongated basal bulb prolonged into a long, spirally coiled copulatory duct with thick walls, ending distally in a sharp extremity: MCO = 40 (38–44; *n* = 6); Ct = 45 (42–47; *n* = 7). Distinct heel, He = 5 (4–6; *n* = 6). Large accessory piece unattached to the basal bulb and pierced distally by the copulatory duct, Ap = 38 (33–39; *n* = 7). Vagina pouch-like in shape, variable in size: V = 29 (19–38; *n* = 6); *v* = 9 (7–11; *n* = 6).

**Diagnosis.** Regarding the morphology of the hook pairs and the vagina, *C. sergemorandi* sp. nov. belongs to the same morphological group as *C. antoineparisellei* sp. nov. (see above). The newly described species possesses a dorsal bar with short auricles, a spirally shaped copulatory duct, and an accessory piece separated from the basal bulb, features observed in many representatives described on tylochromine cichlids such as *C. mulimbwai* and *C. muzumanii*, parasites of the same cichlid host from LT (Muterezi Bukinga et al., 2012), and the non-Tanganyikan *C. berrebii*, C. *kothiasi*, and *C. pouyaudi* from *T. jentinki* (Steindachner, 1894) (Pariselle & Euzet, 1994), and *C. chrysopiformis* and *C. djietoi* from *T. sudanensis* Daget, 1954 (Pariselle, Bitja Nyom & Bilong Bilong, 2014). *C. sergemorandi* sp. nov. is most reminiscent of *C. mulimbwai*. The former differs from *C. mulimbwai* by (i) the shorter dorsal and ventral anchors (respectively, 23–26; 22–24 μm in *C. sergemorandi* sp. nov. vs 32–42; 31–40 μm in *C. mulimbwai*), (ii) the shorter ventral and dorsal bars (respectively, 16–20; 25–27 μm in *C. sergemorandi* sp. nov. vs 33–44; 38–52 μm in *C. mulimbwai*), and (iii) the vagina (no sclerotized vagina in *C. mulimbwai* unlike in *C. sergemorandi* sp. nov.). *C. sergemorandi* sp. nov. differs from *C. muzumanii* by (i) the first hook pair (thin pair I in *C. sergemorandi* sp. nov. vs large pair in *C. muzumanii*), (ii) the shorter dorsal and ventral anchors (respectively, 23–26; 22–24 μm in *C. sergemorandi* sp. nov. vs 44–58; 37–47 μm in *C. muzumanii*), (iii) the shorter dorsal bar and its auricles (respectively, 16–20; 4–5 μm in *C. sergemorandi* sp. nov. vs 45–62; 10–19 μm in *C. muzumanii*), (iv) the shorter ventral bar (25–27 μm in *C. sergemorandi* sp. nov. vs 47–63 μm in *C. muzumanii*), (v) the shorter copulatory duct distally differently shaped (sharp extremity, 42–47 μm in *C. sergemorandi* sp. nov. vs broad extremity (not mentioned in the original description), 57–68 μm in *C. muzumanii*), (vi) the longer accessory piece (33–39 μm in *C. sergemorandi* sp. nov. vs 17–20 μm in *C. muzumanii*), and (vii) the vagina (no sclerotized vagina in *C. muzumanii* vs sclerotized vagina in *C. sergemorandi* sp. nov.).

**Previous record: *Cichlidogyrus* sp. ‘*T. polylepis* 3’** Muterezi Bukinga et al., 2012.

**Type-host:** *Tylochromis polylepis* (Boulenger, 1900) (Perciformes Bleeker, 1859: Cichlidae Heckel, 1840: Tylochromini Poll, 1986).

**Host accession number: MH298008.**

**Site of infection:** Gills.

**Type-locality:** Mulongwe fish market (3°22′S, 29°6′E), Uvira, DRC, LT.

**Prevalence & intensity of infection:** two fish specimens infected/three fish specimens examined, one to five parasite specimens per infected host.

**Holotype**: MRAC M.T.38444.

**Paratype**: MRAC M.T.38445.

**Etymology:** the specific epithet of the new species ‘*sergemorandi*’ honours the French evolutionary biologist and ecologist Dr. Serge Morand from CNRS and CIRAD (France), and CILM (Laos) for his work on ecology and the evolution of parasites.

### Morphological characterisation of the MCO of the undescribed species

**Figure 10:**
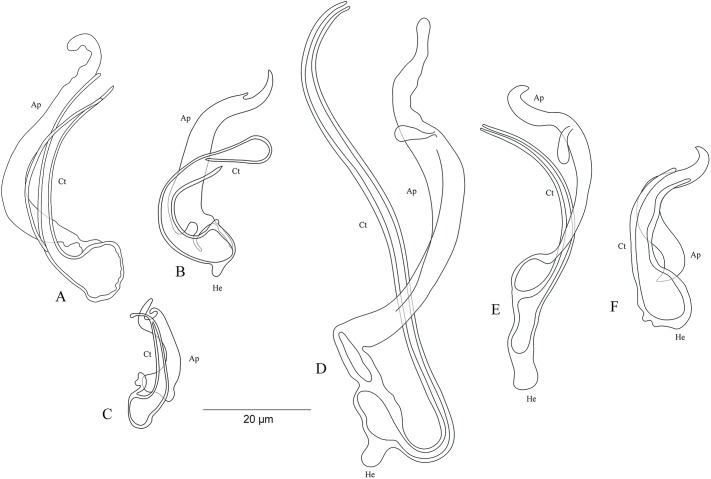
Sclerotized structures of the undescribed species of *Cichlidogyrus* characterized in this study. (A) *Cichlidogyrus* sp. ‘*C. melanostigma*.’ (B) *Cichlidogyrus* sp. ‘*X. flavipinnis* 1.’ (C) *Cichlidogyrus* sp. ‘*X. flavipinnis* 2.’ (D) *Cichlidogyrus* sp. ‘*I. loocki* 5.’ (E) *Cichlidogyrus* sp. ‘*P. orthognathus* 2.’ (F) *Cichlidogyrus* sp. ‘*P. orthognathus* 3.’ He, heel; Ct, copulatory tube; Ap, accessory piece.

On the basis of the MCO features, we characterize six new undescribed species, namely *C.* sp. ‘*C. melanostigma*’, *C.* sp. ‘*X. flavipinnis* 1’, and *C.* sp. ‘*X. flavipinnis* 2’ (all from ectodine hosts), and *C.* sp. ‘*I. loocki* 5’, *C.* sp. ‘*P. orthognathus* 2’, and *C.* sp. ‘*P. orthognathus* 3’ (all from tropheine hosts) (Table 2; Fig. 10A–10F). These species could not be formally described because of the lack of material. Further, the haptoral parts of some specimens were not clearly visible for drawings. However, the general shape of the MCO provided sufficient morphological information to distinguish these species that can be considered as new to science. The morphological characterization of the MCO of the undescribed species of *Cichlidogyrus* recognized on Congolese host specimens did not allow us to confirm the presence/absence of a sclerotized vagina.

**Table 2:** Overview of the undescribed species of *Cichlidogyrus* characterized in the present study with their type-host and locality of sampling.

*Cichlidogyrus* spp.	Host	Type-locality
*C.* sp. ‘*Callochromis melanostigma*’ Fig. 10A	*C. melanostigma* (Boulenger, 1906)	Kilomoni beach (3°20′S, 29°10′E)
*C.* sp. ‘*Xenotilapia flavipinnis* 1’ Fig. 10B	*X. flavipinnis* Poll, 1985	Pemba (3°37′S, 29°9′E)
*C.* sp. ‘*Xenotilapia flavipinnis* 2’ Fig. 10C		
*C.* sp. ‘*Interochromis loocki* 5’ Fig. 10D	*I. loocki* (Poll, 1949)	Pemba (3°37′S, 29°9′E)
*C.* sp. ‘*Petrochromis orthognathus* 2’ Fig. 10E	*P. orthognathus* Matthes, 1959	Pemba (3°37′S, 29°9′E)
*C.* sp. ‘*Petrochromis orthognathus* 3’ Fig. 10F		

***Cichlidogyrus* sp. ‘*C. melanostigma*’ Fig. 10A**

In the gills of the ectodine *C. melanostigma* we found a single monogenean species, namely *C.* sp. ‘*C. melanostigma*’. Its MCO lacks a heel and possesses an ovoid basal bulb prolonged into a curved copulatory duct ending in a large opening: MCO = 46 (45–47; *n* = 2); Ct = 47–48 (*n* = 2). The accessory piece is curved, L-shaped, attached to the basal bulb and ending in a thick hook, Ap = 36–37 (*n* = 2). Further, a visible second wall lines the surface of the copulatory duct, which confers a twisted appearance. *Cichlidogyrus* sp. ‘*C. melanostigma*’ resembles *C. banyankimbonai* described from *S. diagramma* regarding the large distal opening of the copulatory duct (see Van Steenberge et al., 2015). However, the MCO of *C.* sp. ‘*C. melanostigma*’ lacks a heel, unlike that of *C. banyankimbonai*. In addition, *C.* sp. ‘*C. melanostigma*’ exhibits a differently shaped accessory piece ending in a hook, a feature not observed in *C. banyankimbonai*. According to the comparative morphology of the MCO, *C.* sp. ‘*C. melanostigma*’ is reminiscent of *C. discophonum* from *A. dewindti*, both parasites of ectodine cichlids (see Rahmouni, Vanhove & Šimková, 2017). The two species lack a heel and possess a curved copulatory duct, and an accessory piece ending in a hook. However, *C.* sp. ‘*C. melanostigma*’ exhibits a longer accessory piece (36–37 μm in *C.* sp. ‘*C. melanostigma*’ vs 15–22 μm in *C. discophonum*). Further, the copulatory duct in *C.* sp. ‘*C. melanostigma*’ appears double walled and ends in a large opening, while there is a thick proximal part which tapers distally in *C. discophonum*. Also, the accessory piece in *C.* sp. ‘*C. melanostigma*’ is L-shaped, while *C. discophonum* possesses an accessory piece composed of two parts twisted distally. Regarding these differences, we consider *C.* sp. ‘*C. melanostigma*’ to be a different species from *C. discophonum* and new to science.

***Cichlidogyrus* sp. ‘*X. flavipinnis* 1’ Fig. 10B**

The MCO of *C.* sp. ‘*X. flavipinnis* 1’ exhibits a small heel and a thick, well curved copulatory duct, MCO = 37 (*n* = 1); He = 2 (*n* = 1); Ct = 36 (*n* = 1). The subterminal opening of the copulatory duct seems to be located at the last third. Such a feature has never been reported in *Cichlidogyrus* species described so far. Thus, we cannot confirm whether *C.* sp. ‘*X. flavipinnis* 1’ really exhibits this characteristic in its copulatory duct, or it is only an artefact due to isolation or fixation procedures. The accessory piece attached to the basal bulb is thick, curved both proximally and distally with a finger-like ending, Ap = 29 (*n* = 1). *Cichlidogyrus* sp. ‘*X. flavipinnis* 1’ is most similar to *C.* sp. ‘*C. melanostigma*’. Both species occur on closely related ectodine host species, that is, *X. flavipinnis* and *C. melanostigma*. However, *C.* sp. ‘*X. flavipinnis* 1’ presents a heel, a feature missing in *C.* sp. ‘*C. melanostigma*’, in addition to the differently shaped proximal endings of the accessory piece (finger-like in *C.* sp. ‘*X. flavipinnis* 1’ vs thick hook in *C.* sp. ‘*C. melanostigma*’). Therefore, we consider *C.* sp. ‘*X. flavipinnis* 1’ and *C.* sp. ‘*C. melanostigma*’ as two different species.

***Cichlidogyrus* sp. ‘*X. flavipinnis* 2’ Fig. 10C**

The second undescribed species isolated from *X. flavipinnis* has a shorter MCO compared to that of *C.* sp. ‘*X. flavipinnis* 2’, and a slightly curved copulatory duct with a narrow extremity, MCO = 22 (*n* = 1); Ct = 24 (*n* = 1). Heel reduced to inconspicuous, He = 1 (*n* = 1). The accessory piece is proximally thick and irregularly shaped, attached to the basal bulb by a small additional part, distally with pincer-like ending, Ap = 16 (*n* = 1). The heel structure in *C.* sp. ‘*C. melanostigma*’, *C.* sp. ‘*X. flavipinnis* 1’, and *C.* sp. ‘*X. flavipinnis* 2’ is absent to inconspicuous. Further, *C.* sp. ‘*X. flavipinnis* 2’ is easily distinguishable from *C.* sp. ‘*C. melanostigma*’ and *C.* sp. ‘*X. flavipinnis* 1’ (i) by its shorter MCO and a dissimilar copulatory duct with a narrow extremity, and (ii) by the differently sized accessory piece and its characteristic pincer-like shape at its extremity, the distal parts of which are finger and hook-like in *C.* sp. ‘*X. flavipinnis* 1’ and *C.* sp. ‘*C. melanostigma*’, respectively. On the basis of these differences, we consider *C.* sp. ‘*X. flavipinnis* 2’ to be different from *C.* sp. ‘*C. melanostigma*’ and *C.* sp. ‘*X. flavipinnis* 1’ and to represent a new species to science.

***Cichlidogyrus* ‘*I. loocki* 5’ Fig. 10D**

Specimens of *I. loocki* harboured a second parasite species and, according to the morphology of the MCO, we consider it as new to science. *Cichlidogyrus* sp. ‘*I. loocki* 5’ exhibits a long MCO with a heel attached to the side of an irregularly shaped basal bulb, MCO = 79 (*n* = 1); He = 6 (*n* = 1). The copulatory duct of constant width is long, wavy, and well curved proximally, Ct = 104 (*n* = 1). The accessory piece is separated proximally into two thin parts, curved halfway with a twisted gutter-like appearance, distally with an additional small outgrowth and an irregular blunt ending, Ap = 64 (*n* = 1). However, we cannot reliably confirm the blunt shape in the distal part of the accessory piece since we cannot reject the possibility that this feature is an artefact of parasite fixation.

According to the morphology of the MCO, *C.* sp. ‘*I. loocki* 5’ is most similar to *C. georgesmertensi* from *P. babaulti* in having a long and wavy copulatory duct, in addition to a curved accessory piece attached to the basal bulb (see Van Steenberge et al., 2015). *However, C.* sp. ‘*I. loocki* 5’ exhibits a heel attached to the side of the basal bulb like in *C. masilyai* sp. nov., while in *C. georgesmertensi,* it is located at the bottom of the basal bulb as in most species of *Cichlidogyrus*. Further, no gutter-like appearance or additional outgrowth in the distal part of the accessory piece were reported for *C. georgesmertensi. Cichlidogyrus* sp. ‘*I. loocki* 5’ can be compared to *C. vealli* (see Pariselle et al., 2015b). Indeed, the new undescribed species shares the host *I. loocki* with *C. vealli*, but the two parasites were sampled in different localities (northern lakeshore vs southern tip of the lake in the case of *C. vealli*). Moreover, in *C.* sp. ‘*I. loocki* 5’, the extremity of the MCO is narrower than that of *C. vealli*. Also, the undescribed species shows an additional small outgrowth at the proximal third of its MCO, a feature missing in its congener. Further, the accessory piece in *C.* sp. ‘*I. loocki* 5’ presents a gutter-like structure only in the middle part and a blunt extremity. In *C. vealli*, this feature exists throughout the accessory piece. On the basis of these differences, we consider *C.* sp. ‘*I. loocki* 5’ to be a different species from *C. vealli*.

***Cichlidogyrus* sp. ‘*P. orthognathus* 2’ Fig. 10E**

*Cichlidogyrus* sp. ‘*P. orthognathus* 2’ possesses a long MCO exhibiting an elongated irregularly shaped basal bulb with long heel, MCO = 55 (53–57; *n* = 4); He = 7 (6–8; *n* = 4). The copulatory duct is well curved, Ct = 49 (47–51; *n* = 4). The accessory piece is linked to the basal bulb by a thin filament, curved in the middle part, distally with an outgrowth ending in a hook, Ap = 38 (37–40; *n* = 4). *Cichlidogyrus* sp. ‘*P. orthognathus* 2’ presents a morphotype similar to that of its undescribed congener *C.* sp. ‘*I. loocki* 5’, especially in having the additional small outgrowth in the distal part of the accessory piece. However, the MCO and the copulatory duct are shorter in *C.* sp. ‘*P. orthognathus* 2’ than in *C.* sp. ‘*I. loocki* 5’. Further, the heel is located differently (see above). Thus, on the basis of the morphological differences listed above, we separated the species *C.* sp. ‘*P. orthognathus* 2’ from its congener *C.* sp. ‘*I. loocki* 5’.

***Cichlidogyrus* sp. ‘*P. orthognathus* 3’ Fig. 10F**

*Cichlidogyrus* sp. ‘*P. orthognathus* 3’ exhibits a short MCO with a reduced heel, MCO = 33 (31–34; *n* = 2); He = 2–3 (*n* = 2). The copulatory duct is thick, starting in an ovoid basal bulb, curved halfway with a big distal opening, Ct = 30 (28–31; *n* = 2). The accessory piece is linked to the basal bulb, thick and spirally coiled ending in a hook, Ap = 21 (20–22; *n* = 2). The general shape of the MCO of *C.* sp. ‘*P. orthognathus* 3’ is reminiscent of *C. raeymaekersi*, a species described from the tropeine *S. diagramma* (see Van Steenberge et al., 2015). *C. raeymaekersi* shows a short and wide copulatory duct with a poorly developed heel, and a thick spirally coiled accessory piece attached to the basal bulb as observed in *C.* sp. ‘*P. orthognathus* 3’. However, the latter possesses a copulatory duct with an ovoid basal bulb with a distal wide part, different from the elongated bulb and the bevelled ending in *C. raeymaekersi, which makes C.* sp. ‘*P. orthognathus* 3’ distinct from *C. raeymaekersi.*

## Discussion

The cichlid fishes of LT have undergone spectacular diversification, filling a diversity of ecological niches within a short time period, and therefore represent one of the most interesting models of adaptive radiation (Tsuboi et al., 2016). Research on cichlids is nowadays combined with the investigation of their parasite diversity in order to study the speciation of both cichlids and their specific parasites (Vanhove et al., 2015, 2016). Currently, only 32 *Cichlidogyrus* spp. are known from cichlids inhabiting LT (see the overview by Rahmouni et al. (2017)). The present study, based on morphological characters, increases knowledge of the diversity of host specific monogenean species in the cichlids living the northern sub-basin of the lake. We provide descriptions of seven gill monogenean species parasitizing six Congolese representatives of four cichlid tribes in LT. They are *C. adkoningsi* sp. nov. on *C. frontosa* (Cyphotilapiini); *C. koblmuelleri* sp. nov. on *C. schoutedeni* (Ectodini); *C. habluetzeli* sp. nov. on *C. frontosa* and *C. schoutedeni*; *C. antoineparisellei* sp. nov. on *I. loocki*; *C. masilyai* sp. nov. on *P. orthognathus*; *C. salzburgeri* sp. nov. on *P. trewavasae* (both Tropheini); and finally *C. sergemorandi* sp. nov. on *T. polylepis* (Tylochromini). We characterized also six undescribed parasite species on the basis of the morphology of their MCO. They are *C.* sp. ‘*C. melanostigma*’ from *C. melanostigma*; *C.* sp. ‘*X. flavipinnis* 1’ and *C.* sp. ‘*X. flavipinnis* 2’ from *X. flavipinnis* (both Ectodini); *C.* sp. ‘*I. loocki* 5’ from *I. loocki*; and finally *C.* sp. ‘*P. orthognathus* 2’ and *C.* sp. ‘*P. orthognathus* 3’ from *P. orthognathus* (all Tropheini).

First, we identified the cichlid species using morphology and DNA sequence data from the mitochondrial cyt-*b* region. Nowadays, DNA barcoding targeting mitochondrial regions such as the *COI* (cytochrome oxidase I) or cyt-*b* genes for cichlid fish identification is well established and documented (Kullander et al., 2014; Breman et al., 2016). Molecular analyses confirmed the morphological identification of the cichlids analysed for the presence of monogenean parasites in this study.

Then, we morphologically characterized the newly described species of *Cichlidogyrus* on the basis of the sclerotized parts of their attachment (haptor) and reproductive organs (MCO and sclerotized vagina when visible). The sclerotized structures of dactylogyridean monogeneans have been extensively investigated in various ecological and evolutionary contexts. Several studies have reported the influence of such sclerotized structures on host specificity, parasite specialization, and reproductive isolation among congeners through niche ecology (Šimková & Morand, 2008; Vignon, Pariselle & Vanhove, 2011; Messu Mandeng et al., 2015). Because of the limitations of light microscopy as regards some morphological characters, sclerotized structures are increasingly studied using enzymatic digestion followed by scanning electron microscopy. In the case of *Cichlidogyrus*, we can cite recent studies of Fannes, Vanhove & Huyse (2017) and Igeh, Dos Santos & Avenant-Oldewage (2017). Using this method, they redescribed three *Cichlidogyrus* spp. (*C. tiberianus*, *C. dossoui*, and *C. philander*), and provided morphological details on the haptoral and reproductive hard parts, which are not visible with light microscopy.

Regarding our study, most sclerites of *C. adkoningsi* sp. nov. found on *C. frontosa* are reminiscent of species previously described from ectodine cichlids (see diagnosis). Despite these similarities, *C. adkoningsi* sp. nov., which represents the first record of a gill ectoparasite on a Tanganyikan cyphotilapiine, is easily distinguishable from its Tanganyikan congeners by the shape and dimensions of its sclerotized structures (such as the dorsal bar and its very characteristic long auricles, and the MCO with its irregularly shaped heel and S-shaped accessory piece ending in a hook). Similarly, *C. habluetzeli* sp. nov., a common species found on the same host and on the gills of *C. schoutedeni*, exhibits the same morphotype as species infecting ectodines, as well as other *Cichlidogyrus* species described from various Tanganyikan tribes. Morphologically, most of the sclerotized structures of *C. habluetzeli* sp. nov. are bigger than in similar species. Yet, the dorsal bar (with its small hollow outgrowths on the anterior face and its straight auricles), in addition to the straight copulatory duct, heel, and accessory piece, are structures reminiscent of those of *C. aspiralis*, *C. pseudoaspiralis*, *C. centesimus* (Vanhove, Volckaert & Pariselle, 2011; Rahmouni, Vanhove & Šimková, 2017), *C. casuarinus* (Pariselle et al., 2015a), and finally *C. nshomboi* (Muterezi Bukinga et al., 2012). However, some features are present in some species and missing in others. This is the case with the spirally-ornamented wall of the MCO observed exclusively in *C. casuarinus*, *C. centesimus*, and *C. nshomboi*, or the sclerotized vagina present in all the species listed above (including *C. habluetzeli* sp. nov.) except for *C. pseudoaspiralis* (Vanhove, Volckaert & Pariselle, 2011; Muterezi Bukinga et al., 2012; Pariselle et al., 2015a; Kmentová et al., 2016b; Rahmouni et al., 2017). Regarding the haptoral configuration, *C. habluetzeli* sp. nov. with its hook pairs (long pairs I–IV, VI, and VII with large pair I) joins the group that harbours, so far, a single member, *C. centesimus.*

In recent studies on the monogenean diversity of Tanganyikan cichlids, similarities in the haptoral and reproductive organs of *Cichlidogyrus* spp. parasitizing phylogenetically closely related cichlid hosts are far from unusual. In the present study, for instance, *C. habluetzeli* sp. nov. and *C. koblmuelleri* sp. nov. infecting *C. schoutedeni* are morphologically similar to some congeners of ectodine cichlids. This is also true for the undescribed species *C.* sp. ‘*C. melanostigma*’ and *C. discophonum* from *A. dewindti* sampled in north-eastern LT. In fact, Rahmouni et al. (2017) reported that two cichlid hosts *Eretmodus marksmithi* Burgess, 2012 and *Tanganicodus irsacae* Poll, 1950, both belonging to the Eretmodini, host two morphologically similar monogenean species *C. jeanloujustinei* and *C. evikae*, respectively. Moreover, monogenean communities composed of morphologically similar *Cichlidogyrus* species were reported in Burundese *O. nasuta* and its congeners from more southern localities. This observation was explained by the large distribution of ectodine hosts in the lake (Vanhove, Volckaert & Pariselle, 2011; Rahmouni, Vanhove & Šimková, 2017). Also in the parasite fauna of tropheine cichlids, morphologically similar *Cichlidogyrus* species infect phylogenetically related cichlid species (Vanhove, 2012; Pariselle et al., 2015b; Van Steenberge et al., 2015). As reported by Braga, Araújo & Boeger (2014), the phylogenetic relatedness of the host species has a strong influence on the distribution of monogenean parasites. On the basis of molecular data, recent work on Tanganyikan cichlids placed the Ectodini as a sister group to a clade formed by Cyphotilapiini and two other tribes (Eretmodini Poll, 1986 and Haplochromini), all mouthbrooding lineages (Meyer, Matschiner & Salzburger, 2015). This could explain the presence of *C. habluetzeli* sp. nov. on both *C. frontosa* and *C. schoutedeni*. At the same time, Boulengerochromini and Bathybatini are phylogenetically distant from Cyphotilapiini and Ectodini (Meyer, Matschiner & Salzburger, 2015), although they all share the morphotype of *Cichlidogyrus* exhibiting a spiral thickening in the wall of the copulatory duct. Possibly, living in at least partially overlapping habitat(s) could allow unrelated cichlids to host similar *Cichlidogyrus* species. In relation to the unique morphology of the hook pairs observed in *C. habluetzeli* sp. nov. and its congener *C. centesimus*, Rahmouni et al. (2017) already suggested the existence of more haptoral groups in *Cichlidogyrus* than previously reported by Vignon, Pariselle & Vanhove (2011), and proposed a reinvestigation of the structural diversity of the hook pairs in *Cichlidogyrus* spp. and a redefinition of the ‘boundaries’ between the haptoral groups.

In addition to hook morphology, Rahmouni et al. (2017) also discussed the sclerotization of the vagina. On the basis of this study, we find that a few *Cichlidogyrus* spp. described from Tanganyikan hosts and possessing a sclerotized vagina belong to none of the haptoral groups defined by Vignon, Pariselle & Vanhove (2011) (who did not include species of *Cichlidogyrus* from LT (Pariselle & Euzet, 2009; Rahmouni, Vanhove & Šimková, 2017)). This is the case for *C. habluetzeli* sp. nov., *C. casuarinus*, *C. centesimus*, and *C. nshomboi* (Vanhove, Volckaert & Pariselle, 2011; Muterezi Bukinga et al., 2012; Pariselle et al., 2015a). Moreover, only three Tanganyikan species of *Cichlidogyrus* specifically *C. mbirizei* from *O. tanganicae* (Oreochromini) (Muterezi Bukinga et al., 2012) and two of the newly described species (*C. antoineparisellei* sp. nov. from *I. loocki* (Tropheini), and *C. sergemorandi* sp. nov. from *T. polylepis* (Tylochromini)) exhibit short hook pairs I–IV, VI, and VII and a sclerotized vagina. Members of the Oreochromini and Tylochromini tribes occur in the lake but colonized the lake relatively recently (Meyer, Matschiner & Salzburger, 2015). The tribe Oreochromini was proposed only recently (Dunz & Schliewen, 2013). Therefore, formulating hypotheses on the significance of the hooks and the sclerotized vagina, seen together in *Cichlidogyrus* spp., is problematic. Rahmouni et al. (2017) has already underlined the necessity to investigate and analyse whether there is a correlation between the reproductive organs (the presence/absence of a sclerotized vagina) and the haptoral sclerites (the morphology of the hook pairs).

Muterezi Bukinga et al. (2012) reported the presence of *Cichlidogyrus* sp. ‘*T. polylepis* 3’, in addition to two other *Cichlidogyrus* species isolated from *T. polylepis* sampled in the central sub-basin of the lake. Because of the low number of helminth specimens collected and studied, they did not provide a formal description. On the basis of the original drawing of the hard parts of *Cichlidogyrus* sp. ‘*T. polylepis* 3’, we were easily able to identify the species, complete the observations made by Muterezi Bukinga et al. (2012), and formally describe the new species herein as *C. sergemorandi* sp. nov.

The Tropheini is one of the best studied cichlid tribes with respect to ectoparasitic monogeneans. Twelve gill species of *Cichlidogyrus* were previously recognized on eight cichlid species of this tribe (Gillardin et al., 2012; Pariselle et al., 2015b; Van Steenberge et al., 2015). In this study, *I. loocki*, *P. orthognathus*, and *P. trewavasae* sampled from the northern Congolese lakeshore revealed the presence of *C. antoineparisellei* sp. nov., *C. masilyai* sp. nov., and *C. salzburgeri* sp. nov. The latter two species represent the first formal descriptions of gill monogeneans on representatives of *Petrochromis. I. loocki* from the Zambian lakeshore was previously studied for monogenean parasites and three species, *C. buescheri*, *C. schreyenbrichardorum*, and *C. vealli*, were described (Pariselle et al., 2015b). The new species of the present study are morphologically highly differentiated from those described by Pariselle et al. (2015b). Our record is strong new evidence of the geographical variation in parasite diversity in a particular cichlid species throughout LT. Indeed, Vanhove, Volckaert & Pariselle (2011) described four *Cichlidogyrus* spp. from the ectodine host *O. nasuta* and its two congeners *O. ventralis* and *O. boops* sampled along the more southern coastline of LT in DRC, Zambia, and Tanzania, while Rahmouni et al. (2017) reported three different species of *Cichlidogyrus* found on *O. nasuta* inhabiting the northern part of the lake. Moreover, Pariselle et al. (2015b) previously compared the three *Cichlidogyrus* spp. described on *I. loocki* to parasite specimens belonging to hitherto undescribed species retrieved from *Petrochromis* spp. from the collection of the MRAC. They provided micrographs of the haptoral hard parts of potentially six *Cichlidogyrus* spp. isolated from three cichlids (*P. fasciolatus* Boulenger, 1914, a species phylogenetically closely related to *I. loocki*; *P. famula* Matthes & Trewavas, 1960, an intermediate species between the *Petrochromis*/*Interochromis* clade and the ‘large’ *Petrochromis* clade; and finally, *P. trewavasae ephippium* Brichard, 1989, a member of the ‘large’ *Petrochromis*) (Yamaoka, 1997). On the basis of these micrographs, we were able to compare the morphology of the dorsal and ventral anchors and bars and identify differences between the newly described species on representatives of *Petrochromis* and the undescribed species reported by Pariselle et al. (2015b). However, due to the lack of haptoral parts in our specimens (damaged parts), and the lack of reproductive organs in the micrographs of *Cichlidogyrus* included in Pariselle et al. (2015b), we were not able to clearly state whether *Cichlidogyrus* spp. isolated from *P. orthognathus* represents one of the species collected from *P. famula*, *P. fasciolatus*, and *P. trawavasae ephippium*. In contrast, *C. masilyai* sp. nov. and *C. salzburgeri* sp. nov. isolated respectively from *P. orthognathus* and *P. trawavasae* were easily distinguishable from their congeners reported by Pariselle et al. (2015b) by their typical C-shaped dorsal bar with straight auricles and a dorsal bar with relatively wide auricles, respectively.

Regarding the undescribed species of *Cichlidogyrus* presented herein, resampling *C. melanostigma*, *I. loocki*, *P. orthognathus*, and *X. flavipinnis* would allow future researchers to collect more parasite specimens, characterize the haptoral parts, and provide formal descriptions.

## Conclusion

Lake Tanganyika has an impressive degree of cichlid parasite diversity with numerous species as yet undescribed. *Cichlidogyrus* includes (to date) 39 species from LT, including the new species described in the present study, and 79 species from other locations. Species of *Cichlidogyrus* infecting closely related cichlid fishes are often morphologically similar, but taxonomically distinct. Further morphological and molecular studies are necessary to elucidate the origin of the various lineages of *Cichlidogyrus* in LT and their evolutionary history.

## Institutional Abbreviations

MNHN: Muséum National d’Histoire Naturelle, Paris, France.
MZH: Finish Museum of Natural History, Helsinki, Finland.
MRAC: Royal Museum for Central Africa, Tervuren, Belgium.

## Additional Information and Declarations

### Field Study Permissions

The following information was supplied relating to field study approvals (i.e. approving body and any reference numbers): Sampling was carried out under mission statements 022/MINEURS/CRH-U/2013 and 031/MINRST/CRH-U/2016 from the Centre de Recherche en Hydrobiologie-Uvira. In the absence of relevant animal welfare regulations in the D.R. Congo, the same strict codes of practice enforced within the European Union were applied.

### DNA Deposition

The following information was supplied regarding the deposition of DNA sequences: GenBank: MH297985–MH298008.

### New Species Registration

The following information was supplied regarding the registration of a newly described species: Publication LSID: urn:lsid:zoobank.org:pub:7076794A-B9EB-4FFC-AC49-66C304EC5BFB. *Cichlidogyrus adkoningsi* sp. nov. LSID urn:lsid:zoobank.org:act:526C2A74-E3D6-4357-B1E8-03B08B95CE38. *Cichlidogyrus koblmuelleri* sp. nov. LSID urn:lsid:zoobank.org:act:473DB764-6798-43EE-8DD8-3B10D1AC1BBF. *Cichlidogyrus habluetzeli* sp. nov. LSID urn:lsid:zoobank.org:act:EC79CE69-2D88-4D96-A255-AC0E31B76EAE. *Cichlidogyrus antoineparisellei* sp. nov. LSID urn:lsid:zoobank.org:act:B324AD44-4520-4129-8DBB-8985A981783D. *Cichlidogyrus masilyai* sp. nov. LSID urn:lsid:zoobank.org:act:4D5AF9E3-9CCE-4D3F-95B4-CCDA7B1A5C91. *Cichlidogyrus salzburgeri* sp. nov. LSID urn:lsid:zoobank.org:act:612A6D2B-09DD-4902-8640-A4877277E7F3. *Cichlidogyrus sergemorandi* sp. nov. urn:lsid:zoobank.org:act:7A4653B3-2C25-4E3A-9D4A-3FA76165C167.

### Data Availability

The following information was supplied regarding data availability: The type-material of the new species described in this study is housed in the invertebrate collection of the Royal Museum for Central Africa (MRAC), Tervuren, Belgium; the Finnish Museum of Natural History (MZH), Helsinki, Finland; and the Muséum National d’Histoire Naturelle (MNHN), Paris, France (see taxonomic summaries for details on repositories and accession numbers).
